# Multi-omics-guided targeting of the CD14–NF-κB axis with PGA1-loaded liposomes restores CD14^hi^ TSPCs function and promotes tendon–bone healing in diabetes

**DOI:** 10.1016/j.mtbio.2026.103509

**Published:** 2026-07-30

**Authors:** Minghao Tong, Liyan Liu, Wanshun Liu, Haocheng Qin, Zhixuan Mai, Pingkang Qian, Yinhua Qian, Haoqiang Huang, Quan Yang, Xiaofeng Wu, Chen Kuang, Yanwei He, Renwen Wan, Wei Luo, Yuxi Ou, Feng Xu, Xiaolan Cheng, Zhiwen Luo, Qing Wang

**Affiliations:** a Department of Orthopedics, Kunshan Hospital of Chinese Medicine, Kunshan Hospital Affiliated to Nanjing University of Chinese Medicine, No.388 Zuchongzhi South Road, Kunshan, Jiangsu, 215300, China; b Department of Endocrinology, Kunshan Hospital of Chinese Medicine, Kunshan Hospital Affiliated to Nanjing University of Chinese Medicine, No.388 Zuchongzhi South Road, Kunshan, Jiangsu, 215300, China; c Department of Sports Medicine, Nanjing Hospital of Chinese Medicine, Nanjing University of Chinese Medicine, China; d Department of Rehabilitation, Huashan Hospital, Fudan University, Shanghai, 200040, China; e Department of Sports Medicine, Huashan Hospital, Fudan University, Shanghai, 200040, China; f Department of Urology, Huashan Hospital, Fudan University, Shanghai, 200040, China; g School of Integrated Chinese and Western Medicine, Nanjing University of Chinese Medicine, No. 138 Xianlin Avenue, Qixia District, Nanjing, Jiangsu, 210023, China; h Fudan University-Dr. Kong Joint Research Center for Sports Medicine and Health Footwear, Fudan University Institute of Sports Medicine (Jinqiao Laboratory), Zhangjiang Institute, Fudan University, Shanghai, China

**Keywords:** CD14, PGA1, TSPCs, Diabetes, Tendon–bone healing, Rotator cuff tear, Liposomes, Multi-omics

## Abstract

**Background:**

Type 2 diabetes mellitus (T2DM) is common in patients with rotator cuff tears (RCTs) and is associated with poor tendon–bone healing and high retear rates. Tendon stem/progenitor cells (TSPCs) are central to tendon–bone integration, but how T2DM reprograms TSPCs and whether this can be reversed remain unclear.

**Methods:**

T2DM was induced in rodents, followed by tendon single-cell RNA sequencing, serum metabolomics and tendon proteomics. TSPCs were isolated for functional assays and analysis of underlying signaling. CD14-overexpressing (CD14^hi^) TSPCs modeled diabetic-like dysfunction. Free PGA1 and Lipo@PGA1 were evaluated in vitro and in a diabetic RCT model, alongside liposome characterization and safety assessment.

**Results:**

Multi-omics analysis revealed that T2DM induced marked remodeling of the tendon-resident cell landscape, with TSPCs exhibiting upregulated CD14 expression and enrichment of NF-κB–related inflammatory and apoptotic pathways, alongside systemic depletion of serum PGA1. Diabetic and CD14^hi^ TSPCs showed reduced progenitor-like functional properties, accompanied by activation of the CD14-NF-κB axis. PGA1 supplementation or CD14 silencing attenuated NF-κB activation and restored TSPC stemness. PGA1-loaded liposomes showed favorable physicochemical properties, good local retention at the tendon–bone interface, and acceptable systemic and cellular biocompatibility. In the diabetic RCT model, Lipo@PGA1 significantly increased TSPCs, improved tendon–bone healing, and enhanced biomechanical properties and forelimb function.

**Conclusion:**

T2DM reprograms tendon-resident TSPCs through a CD14–PGA1–NF-κB axis, driving apoptosis and reducing progenitor-like functional properties. Multi-omics-guided targeting of this axis with PGA1-loaded liposomes restores CD14^hi^ TSPCs function and enhances tendon–bone repair in diabetic RCT, supporting a mechanism-based therapeutic strategy.

## Graphical abstract

**Schematic depiction.** Schematic summary of the multi-omics–guided mechanism by which PGA1-loaded liposomes restore TSPC function and promote tendon-bone healing in type 2 diabetes.

**Figure:**
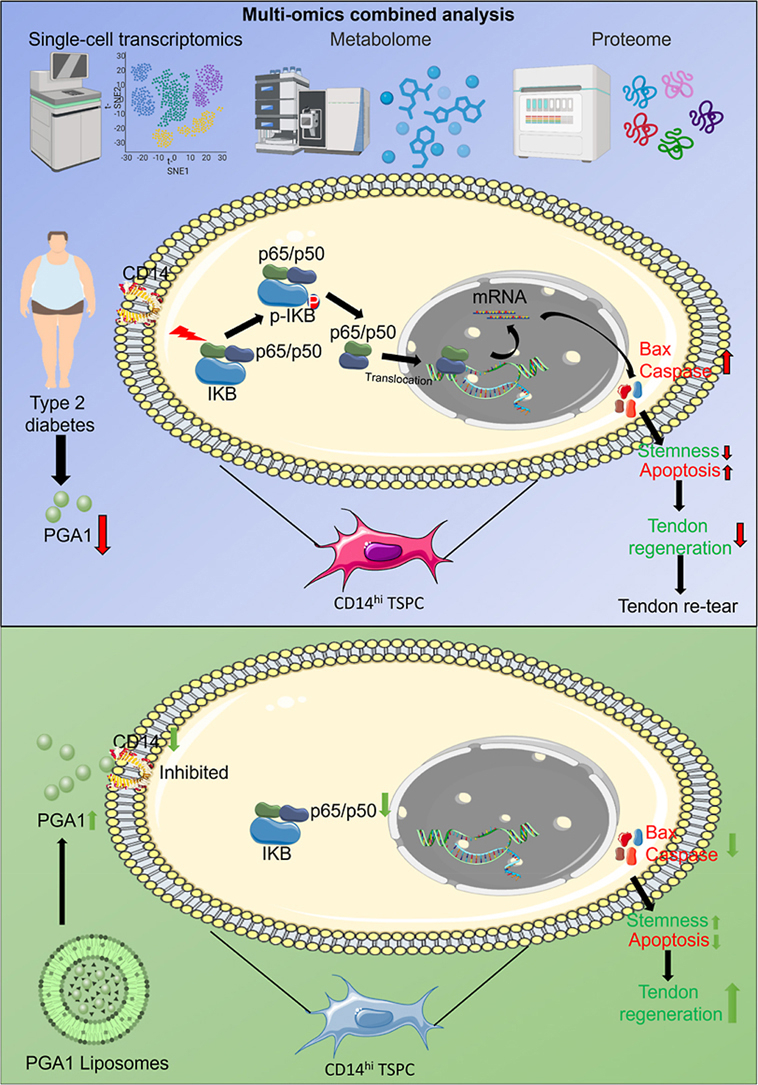

## 1 Introduction

Rotator cuff tears (RCTs) are among the most common causes of shoulder pain and disability in the aging population, and the demand for rotator cuff repair continues to rise with global population aging and increasing longevity [1,2]. Open or arthroscopic rotator cuff repair can effectively relieve pain and improve shoulder function; however, high rates of structural failure and retears after surgery remain a major clinical challenge. Traditional risk factors for retears include large tear size, tendon retraction, fatty infiltration, older age, low bone mineral density, smoking, and poor tissue quality at the tendon–bone interface [[3], [4], [5]]. Type 2 diabetes mellitus (T2DM) is highly prevalent among patients undergoing rotator cuff repair and has emerged as an independent risk factor for both primary RCTs and postoperative retearing, with diabetics showing inferior tendon–bone healing and worse functional outcomes compared with non-diabetic patients [[6], [7], [8], [9], [10]]. Hyperglycemia, advanced glycation end-product (AGE) accumulation, microangiopathy and chronic low-grade inflammation are thought to impair tendon matrix homeostasis and enthesis biology in diabetes [[10], [11], [12], [13]]. Nevertheless, there is still no disease-modifying therapy specifically targeting the diabetic tendon microenvironment, and current clinical protocols have limited ability to restore robust tendon–bone integration in this high-risk population [[14], [15], [16]].

Tendon stem/progenitor cells (TSPCs) are a specialized population of resident stromal cells that play a pivotal role in tendon homeostasis and tendon–bone healing due to their self-renewal capacity and multilineage differentiation potential [[17], [18], [19]]. TSPCs reside within the tendon proper and at the enthesis, where they contribute to maintenance and repair of the tendon structure by generating extracellular matrix (ECM), supporting fibrocartilage formation and orchestrating cell–matrix remodeling [[20], [21], [22]]. The preservation of TSPC “stemness”-encompassing self-renewal, controlled proliferation, and the ability to differentiate into tenocytes and fibrocartilage-like cells -is critical for effective tendon regeneration and for re-establishing a graded tendon–bone interface after injury [18,19,23]. Disruption of TSPC function, whether through aging, osteoporosis or metabolic diseases, leads to diminished regenerative capacity, aberrant ECM deposition, increased apoptosis and a higher propensity for tendon degeneration and retearing [24,25]. Emerging evidence suggests that chronic inflammatory and metabolic cues, including activation of NF-κB and other stress pathways, can drive tendon-resident progenitors toward a senescent or pro-apoptotic, low-stemness state, thereby linking systemic comorbidities such as T2DM with local failure of tendon–bone healing [26,27].

To enhance the regenerative potential of TSPCs and promote tendon–bone healing, nanobiomaterials have attracted considerable attention due to their ability to modulate stem cell behavior at molecular and microenvironmental levels [[28], [29], [30]]. Among these, liposomes are particularly attractive because their phospholipid bilayer closely mimics natural cell membranes, enabling efficient interaction with cells and controlled delivery of bioactive agents to specific stromal or progenitor populations [[31], [32], [33]]. Liposomes can encapsulate both hydrophilic and hydrophobic cargos, protect labile molecules such as siRNAs or lipid mediators from degradation, and provide sustained release at the site of injury [[33], [34], [35]]. Their surfaces can be functionalized to enhance cell-specific targeting, thus improving on-target efficacy while minimizing off-target effects and systemic toxicity [36,37]. In the context of tendon–bone healing, liposomal carriers offer the opportunity to locally deliver small molecules, nucleic acids or proteins that preserve TSPC stemness, modulate inflammatory signaling and promote fibrocartilage formation at the enthesis. We have previously validated this concept in an osteoporosis-related RCT model, where we identified a CD248^+^, low-stemness TSPC subcluster and used si-CD248–loaded liposomes to restore TSPC stemness, enhance tendon–bone integration, and improve postoperative shoulder function [25], underscoring the potential of bioactive liposomes to target disease-specific TSPC subsets.

Building on those works, the present study aimed to define how T2DM reprograms tendon-resident TSPCs and to develop a mechanism-based biomaterial strategy to rescue their dysfunction. Using an integrated multi-omics approach, we identified a CD14–NF-κB inflammatory axis together with systemic depletion of the pro-resolving lipid mediator PGA1 as key features linking diabetes to TSPC apoptosis, stemness loss, and impaired tendon regeneration. Guided by these findings and molecular docking/dynamics predicting a possible interaction between PGA1 and CD14, we engineered PGA1-loaded liposomes for early local delivery to the tendon–bone interface, and evaluated them in CD14^hi^ TSPC models and a diabetic rat supraspinatus RCT model. Our goal was to restore CD14^hi^ TSPCs function through multi-omics-guided targeting of the CD14–NF-κB axis with PGA1-loaded liposomes and to establish a translatable strategy for improving tendon–bone healing in diabetes.

## 2 Results

### 2.1 Multi-omics profiling of diabetic tendons identifies CD14 upregulation in TSPCs and systemic depletion of PGA1

We first confirmed successful establishment of type 2 diabetes in mice and rats, as evidenced by significantly increased body weight and markedly elevated fasting blood glucose (FBG) in diabetic animals compared with normoglycemic controls (Supplementary Fig. 1, Supplementary Fig. 6).

**Fig. 1:**
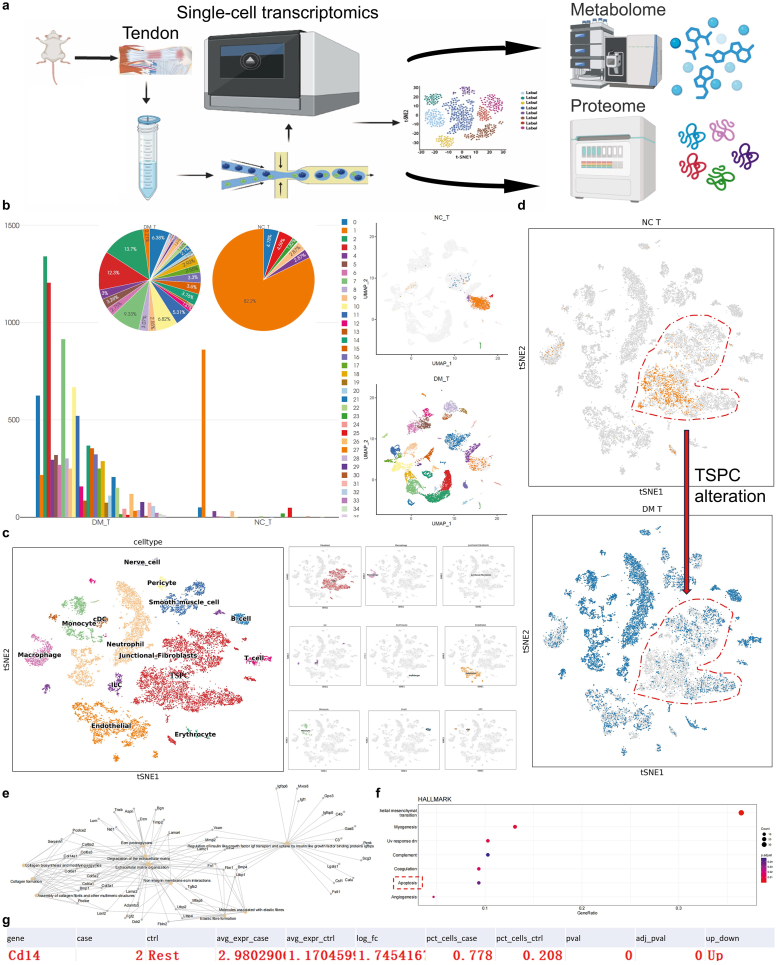
**Single-cell RNA sequencing of Achilles tendon reveals altered tendon stem/progenitor cell (TSPC) landscape and CD14 upregulation in type 2 diabetic mice.** (a) Schematic illustration of the experimental workflow: tendons were harvested from normoglycemic control (NC) and type 2 diabetic (DM) mice, enzymatically digested into single cells and subjected to single-cell RNA sequencing, with downstream integration with bulk proteomic and metabolomic profiling. (b) Bar and pie charts showing the distribution and relative proportion of major tendon-derived clusters in NC_T and DM_T groups. (c) UMAP visualization of tendon-derived single cells after unsupervised clustering. Initial transcriptomic clusters were subsequently assigned to major tendon-resident cell populations according to canonical tendon and stromal marker genes. (d) t-SNE visualization showing the annotated tendon-resident cell populations or TSPC-enriched compartment in NC_T and DM_T tendons. Representative marker genes used for cluster identification are shown in Supplementary Fig. S3. (e) Functional sub-clustering of TSPCs based on gene expression signatures, showing distinct TSPC states enriched for stemness-related, inflammatory, matrix-remodeling and stress-response programs. (f) KEGG pathway enrichment analysis of differentially expressed genes in diabetic versus control TSPCs, indicating activation of inflammatory and NF-κB–related pathways and suppression of stemness-associated signaling. (g) Focused summary of CD14 differential expression in diabetic versus control TSPCs. CD14 was prioritized from the differential-expression analysis and was highlighted as a diabetes-associated inflammatory receptor candidate.

High-quality single-cell RNA sequencing (scRNA-seq) libraries were generated from tendons of control (NC_T) and diabetic (DM_T) mice. Quality-control filtering based on gene number, UMI counts and mitochondrial read percentage yielded comparable data quality across samples (Supplementary Fig. 2). Unsupervised clustering was first performed to define transcriptomic clusters, followed by cell-type annotation based on canonical tendon and stromal marker genes. The annotated tendon-resident populations included TSPC-enriched progenitor-like stromal cells, fibroblasts, endothelial cells, pericytes, immune cells and other stromal populations (Fig. 1b and c; Supplementary Fig. S3a). Diabetes induced a clear redistribution of these annotated populations, with a pronounced remodeling of the TSPC compartment (Fig. 1b–d).

Subclustering of TSPCs revealed several functional states characterized by distinct transcriptional programs related to stemness, extracellular matrix (ECM) remodeling, inflammatory response and stress signaling (Fig. 1e; Supplementary Fig. 3b and c). Differential gene-expression and KEGG enrichment analyses demonstrated that TSPCs from diabetic tendons were enriched for inflammatory and NF-κB–related pathways and showed downregulation of genes associated with stemness maintenance and matrix homeostasis (Fig. 1f; Supplementary Fig. 3b and c). Differential-expression analysis identified that CD14 was significantly upregulated in diabetic TSPCs compared with controls (Fig. 1g). This finding supported CD14 as a key diabetes-associated receptor in the tendon stem/progenitor compartment.

**Fig. 2:**
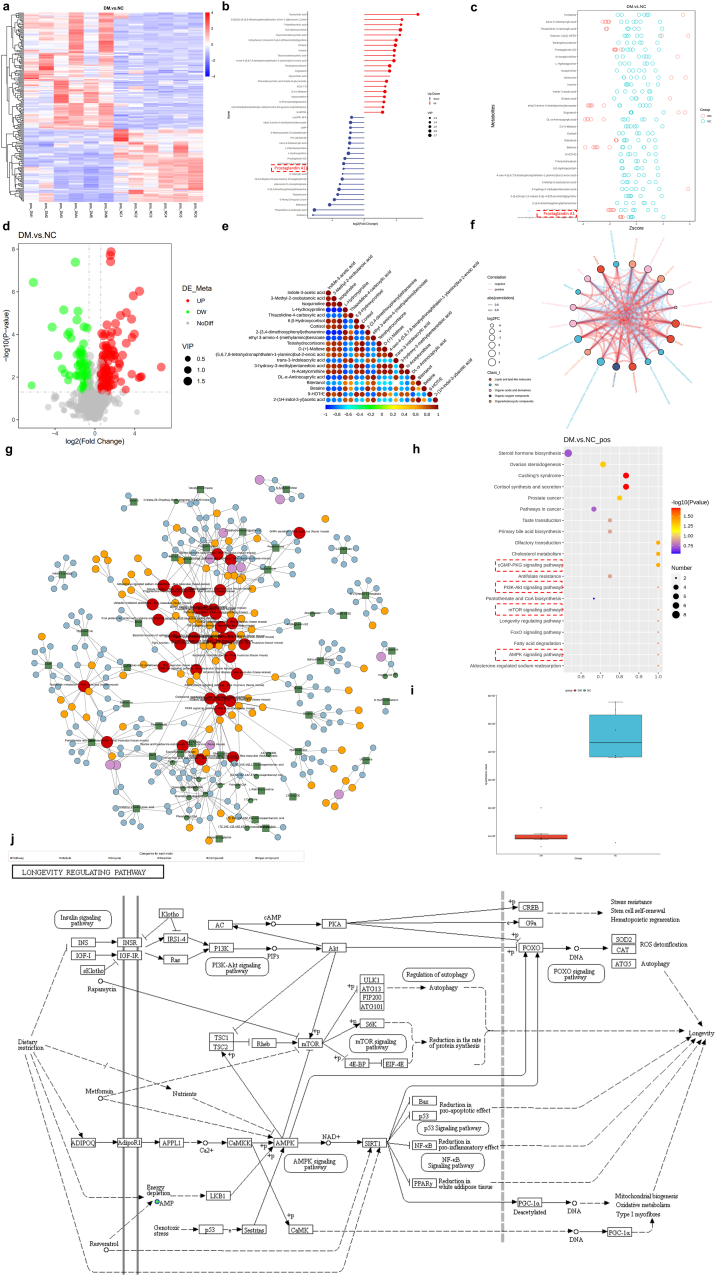
**Serum metabolomic profiling identifies dysregulated metabolic pathways and reduced PGA1 levels in type 2 diabetic mice**. (a) Heatmap of differentially abundant serum metabolites between normoglycemic control (NC) and diabetic (DM) mice, showing unsupervised hierarchical clustering of samples and metabolites according to normalized abundance values. (b) Ranked plot of representative significantly altered metabolites, displaying variable importance in projection (VIP) scores and fold changes for key discriminative metabolites between NC and DM groups. (c) Dot plot illustrating the distribution of selected differential metabolites in NC versus DM serum, with individual metabolite Z-scores indicating direction and magnitude of change. (d) Volcano plot of the metabolomic comparison (DM vs. NC), highlighting significantly upregulated (green) and downregulated (red) metabolites based on log2(fold change) and –log10(p value) thresholds. (e) Correlation matrix of major differential metabolites, depicting pairwise Pearson correlation coefficients and revealing metabolite clusters with highly coordinated changes in diabetes. (f) Metabolite–metabolite interaction network derived from correlation and pathway information, visualizing hubs and modules potentially involved in the diabetic metabolic phenotype. (g) Pathway enrichment bubble plot of differential metabolites, summarizing over-represented metabolic pathways and their enrichment ratios, with dot size and color indicating the number of hits and statistical significance, respectively. (h-i) KEGG pathway enrichment analysis of positively ionized differential metabolites (DM vs. NC_pos), emphasizing enrichment of steroid hormone biosynthesis, arachidonic acid metabolism and related lipid pathways, together with quantitative comparison of serum PGA1 levels demonstrating a marked reduction in PGA1 in diabetic mice. (j) KEGG “Longevity regulating pathway” map showing the upstream insulin/IGF, AMPK, mTOR and FOXO signaling cascades, with altered diabetes-related metabolites and nodes indicated, suggesting that the observed metabolic reprogramming in diabetic mice is linked to longevity-associated regulatory networks.

To understand systemic metabolic changes associated with tendon pathology, we performed untargeted serum metabolomics in NC and diabetic mice. Differential metabolites clearly segregated the two groups on hierarchical clustering and ranked VIP plots (Fig. 2a–c). Volcano and correlation analyses identified coordinated alterations in multiple lipid and energy-related metabolites (Fig. 2d–f). Pathway enrichment highlighted dysregulation of steroid hormone biosynthesis, arachidonic acid metabolism and related lipid pathways (Fig. 2g and h). Notably, the pro-resolving lipid mediator PGA1 was significantly reduced in diabetic serum (Fig. 2i), suggesting that systemic PGA1 deficiency may contribute to chronic inflammation and impaired tendon repair. Mapping differential metabolites onto the KEGG “longevity regulating pathway” further linked the diabetic metabolome to perturbed insulin/IGF, AMPK, mTOR and FOXO signaling axes (Fig. 2j). These pathways were considered to reflect the systemic metabolic background of diabetes. To identify a local mechanism more directly related to tendon-resident progenitor-like cell dysfunction, we integrated the metabolomic results with tendon scRNA-seq and proteomic data. CD14 was then prioritized because it was upregulated in diabetic TSPC-enriched cells and was associated with inflammatory and apoptotic changes in diabetic tendons.

**Fig. 3:**
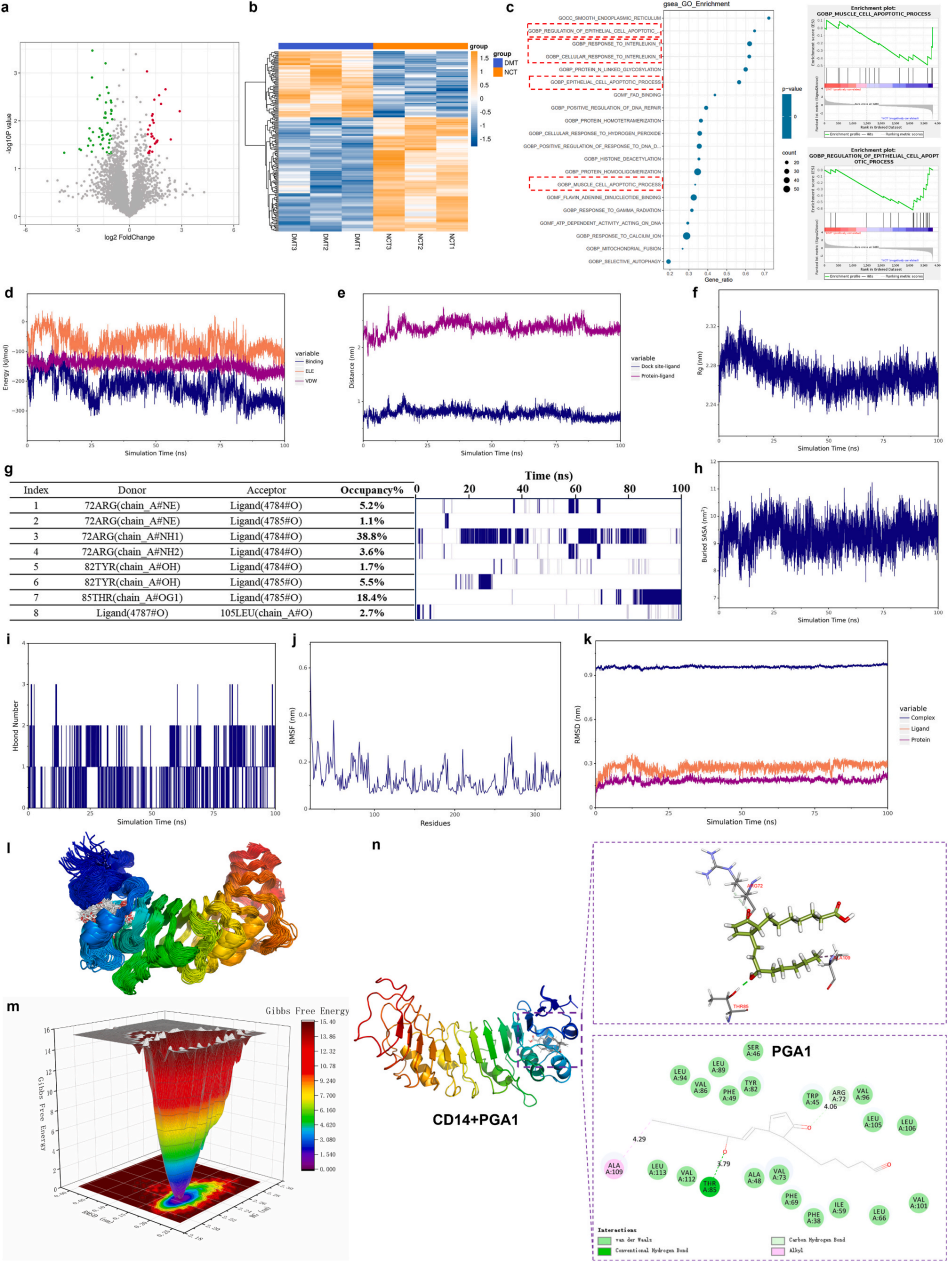
**Tendon proteomic profiling in diabetic mice and molecular docking/MD simulations identify CD14 as a PGA1-binding target**. (a) Volcano plot of differentially expressed proteins in Achilles tendons from diabetic (DM_T) versus normoglycemic control (NC_T) mice, with significantly upregulated and downregulated proteins highlighted according to log2(fold change) and –log10(adjusted p value). (b) Unsupervised hierarchical clustering heatmap of significantly altered proteins, showing distinct expression patterns between NC_T and DM_T samples. (c) Gene Ontology (GO) enrichment analysis of differentially expressed proteins, with a dot plot summarizing over-represented biological processes, and representative GSEA enrichment plots for pathways of interest related to inflammatory response, cell apoptosis and extracellular matrix organization. (d–n) Molecular docking and molecular dynamics (MD) simulation analyses predicting a putative interaction between CD14 and the candidate metabolite PGA1. (d) Time-dependent profile of total binding energy and its electrostatic (ELE) and van der Waals (VDW) components during a 100-ns MD simulation. (e) Time evolution of the distance between PGA1 and key residues in the CD14 binding pocket and the center-of-mass distance between ligand and protein. (f) Radius of gyration (Rg) of CD14 indicating overall compactness of the protein during simulation. (g) Temporal contact map showing the persistence of residue–ligand contacts across the MD trajectory. (h) Solvent-accessible surface area (SASA) of the protein–ligand complex as a function of time. (i) Number of hydrogen bonds formed between CD14 and PGA1 throughout the simulation. (j) Residue-wise root-mean-square fluctuation (RMSF) profile of CD14, highlighting flexible versus rigid regions in the binding environment. (k) Root-mean-square deviation (RMSD) of the protein, ligand and protein–ligand complex, demonstrating overall structural stability of the docked complex. (l) Three-dimensional cartoon representation of the equilibrated CD14–PGA1 complex. (m) Free-energy landscape of the complex derived from principal component analysis, illustrating the dominant low-energy conformational basin of the bound state. (n) Representative docking pose of PGA1 within the CD14 binding pocket and a 2D interaction diagram summarizing hydrogen bonds and hydrophobic contacts between PGA1 and surrounding residues.

Proteomic profiling of Achilles tendons corroborated the transcriptional data. Diabetic tendons exhibited extensive changes in ECM, inflammatory and apoptosis-related proteins compared with controls (Fig. 3a and b). GO and GSEA analyses underscored enrichment of pathways associated with inflammatory response, cell death and ECM organization (Fig. 3c), consistent with a degenerative microenvironment. Integrating the metabolomic and proteomic findings, we focused on CD14 as a putative receptor for PGA1. Molecular docking followed by extensive molecular dynamics simulations predicted a stable binding mode between PGA1 and the CD14 binding pocket, with favorable binding energies, persistent hydrogen bonding and a single dominant low-energy basin on the free-energy landscape (Fig. 3d–m). The final equilibrated complex and interaction maps highlighted specific residues mediating PGA1–CD14 contacts (Fig. 3l–n), suggesting a possible structural link between PGA1 depletion and CD14-associated inflammatory signaling in TSPCs.

### 2.2 TSPC dysfunction and CD14–NF-κB–mediated loss of stemness in diabetes

**Fig. 4:**
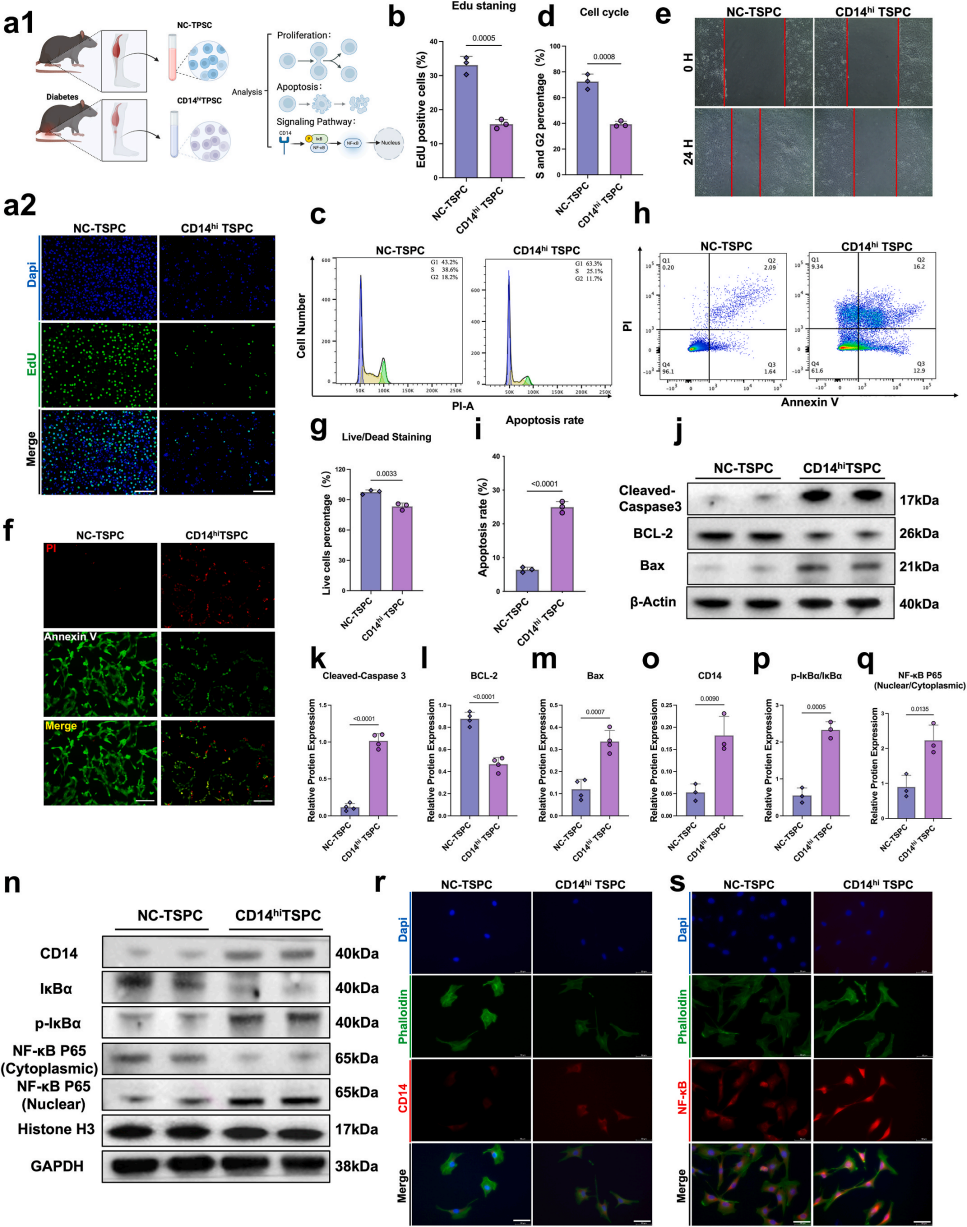
**Functional impairment of tendon-derived TSPCs from diabetic mice and involvement of the CD14–NF-κB axis.** (a, b) EdU incorporation assay showing proliferative activity of TSPCs isolated from control and diabetic tendons under the indicated conditions; representative fluorescence images (a) and quantitative analysis of EdU-positive nuclei (b). Scale bar = 200 μm. (c, d) Cell-cycle distribution of TSPCs assessed by flow cytometry, illustrating altered proportions of cells in G0/G1, S and G2/M phases in diabetic versus control/treated groups, consistent with impaired proliferative capacity. (e) Representative images and quantification of TSPC migration in the scratch-wound assay, demonstrating reduced migratory ability in diabetic TSPCs and its modulation by the indicated treatments. (f, g) Flow-cytometric analysis of apoptosis using Annexin V/PI double staining, with representative dot plots (f) and statistical summary of early and late apoptotic cells (g). Scale bar = 200 μm. (h, i) Live/dead fluorescence staining of TSPCs, showing overall cell viability and increased cell death in diabetic TSPCs compared with controls, together with semi-quantitative analysis. (j–m) Western blot analysis of apoptosis-related proteins in TSPCs, including cleaved caspase-3, Bax, Bcl-2 and other indicated markers; representative blots (j–l) and densitometric quantification normalized to loading controls (m) confirm enhanced apoptotic signaling in diabetic TSPCs. (n–q) Western blot analysis of CD14 and downstream NF-κB signaling components in TSPCs, showing upregulation of CD14, increased phosphorylation of IκBα and p65, and corresponding quantification across groups, indicating activation of the CD14–NF-κB pathway in diabetic TSPCs. (r, s) Immunofluorescence staining for CD14 (r) and NF-κB (s) in tendon-derived TSPCs, with DAPI-labeled nuclei and phalloidin-labeled F-actin, demonstrating enhanced CD14 expression and nuclear/cytoplasmic NF-κB accumulation in diabetic TSPCs and providing semi-quantitative spatial validation of CD14–NF-κB pathway activation. Scale bar = 100 μm.

To validate the scRNA-seq findings at the functional level, we isolated TSPCs from control and diabetic mouse tendons and performed a series of cellular assays. Diabetic TSPCs displayed significantly reduced EdU incorporation and altered cell-cycle distribution, with accumulation in G0/G1 and reduced S-phase entry, indicating impaired proliferative capacity (Fig. 4a–d). Scratch-wound assays further showed diminished migratory ability in diabetic TSPCs compared with controls (Fig. 4e).

Apoptosis analyses demonstrated a clear survival deficit in diabetic TSPCs. Annexin V/PI staining revealed increased proportions of early and late apoptotic cells (Fig. 4f and g), which was corroborated by live/dead staining showing a higher fraction of non-viable cells (Fig. 4h and i). Consistently, Western blotting confirmed upregulation of pro-apoptotic proteins (e.g., cleaved caspase-3, Bax) and downregulation of anti-apoptotic Bcl-2 in diabetic TSPCs (Fig. 4j–m).

Mechanistically, diabetic TSPCs expressed markedly higher levels of CD14, accompanied by increased phosphorylation of IκBα and p65, indicative of NF-κB pathway activation (Fig. 4n–q). Immunofluorescence staining further demonstrated enhanced CD14 signal and nuclear/cytoplasmic NF-κB accumulation in diabetic TSPCs (Fig. 4r and s), in line with the transcriptional and proteomic data (Fig. 1, Fig. 3). Together, these results establish that diabetes drives a CD14–NF-κB–dependent shift of TSPCs toward an apoptotic, low-stemness state.

### 2.3 PGA1 and si-CD14 restored TSPC dysfunction

**Fig. 5:**
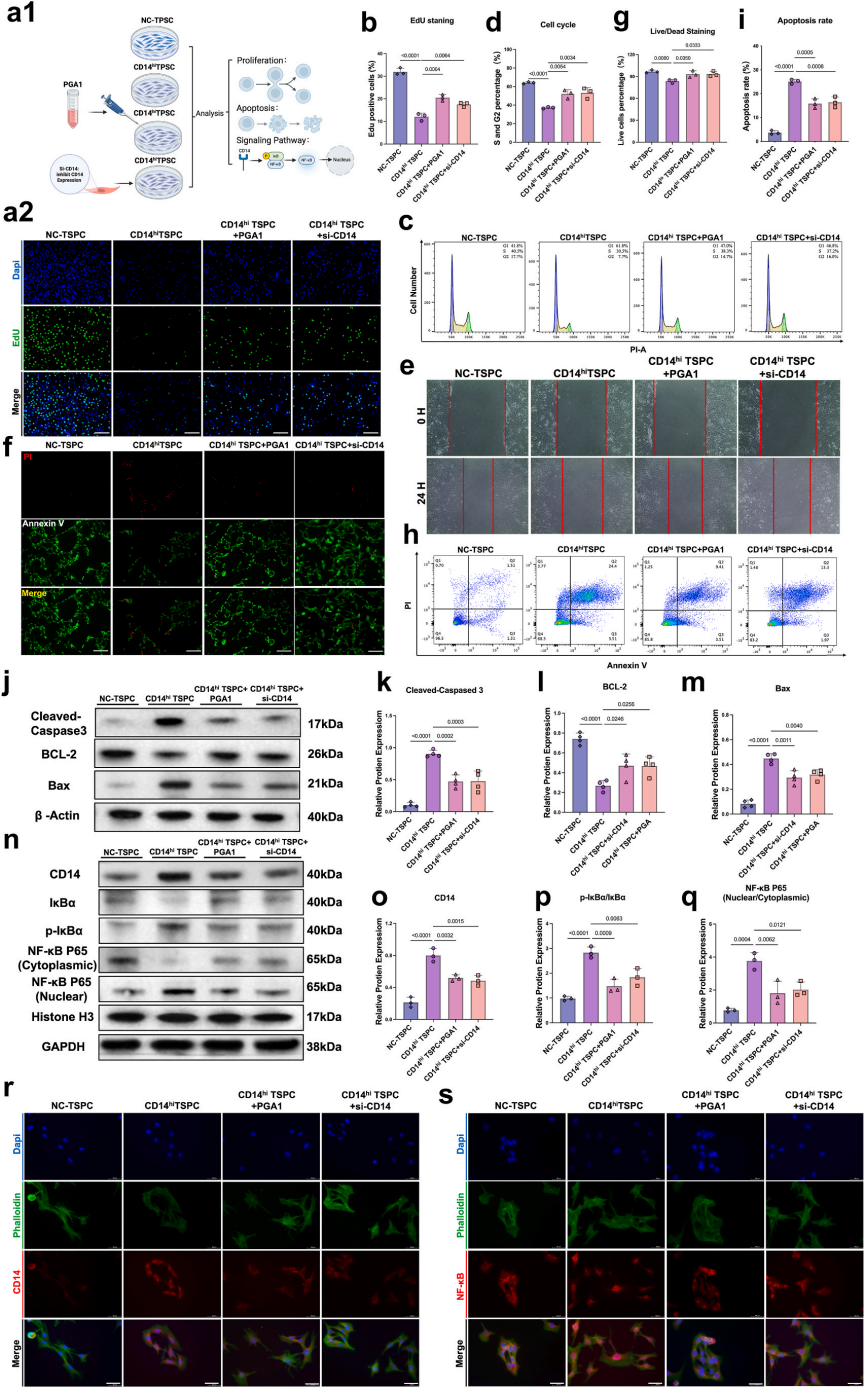
**PGA1 treatment or CD14 silencing reverses CD14-driven dysfunction of tendon-derived TSPCs.** (a, b) EdU incorporation assay in TSPCs from the four groups (NC-TSPC, CD14^hi^ TSPC, CD14^hi^ TSPC + PGA1, and CD14^hi^ TSPC + si-CD14), with representative fluorescence images (a) and quantitative analysis of EdU-positive nuclei (b), showing that CD14 overexpression suppresses proliferation, which is partially rescued by PGA1 treatment and by CD14 knockdown. Scale bar = 200 μm. (c, d) Flow-cytometric analysis of cell-cycle distribution, illustrating G0/G1 arrest and reduced S-phase fraction in CD14^hi^ TSPCs, while PGA1 or CD14siRNA restores cell-cycle progression toward a pattern similar to NC-TSPCs. (e) Scratch-wound migration assay with representative phase-contrast images at the indicated time points, demonstrating impaired migratory capacity in CD14^hi^ TSPCs and improved wound closure after PGA1 treatment or CD14 silencing. (f, g) Live/dead fluorescence staining of TSPCs in each group and corresponding quantification, indicating decreased viability and increased dead cell proportion in CD14^hi^ TSPCs, which are markedly ameliorated by PGA1 or CD14siRNA. Scale bar = 200 μm. (h, i) Flow-cytometric apoptosis analysis using Annexin V/PI double staining, with dot plots (h) and statistical summary (i) of early and late apoptotic cells, showing that CD14 overexpression enhances apoptosis, whereas PGA1 treatment and CD14 knockdown significantly reduce apoptotic rates. (j–m) Western blot analysis of apoptosis-related proteins (e.g., cleaved caspase-3, Bax, Bcl-2 and other indicated markers) across the four groups, with representative blots (j–l) and densitometric quantification (m), confirming that PGA1 and CD14-siRNA correct the pro-apoptotic/anti-apoptotic imbalance induced by CD14 overexpression. (n–q) Western blot analysis of CD14 and downstream NF-κB signaling components, including CD14, p-IκBα, total IκBα, p-p65 and total p65, demonstrating that PGA1 treatment and CD14 silencing attenuate CD14 expression and NF-κB activation in CD14^hi^ TSPCs. (r, s) Immunofluorescence staining for CD14 (r) and NF-κB (s) in TSPCs from the four groups, with DAPI-labeled nuclei and phalloidin-labeled F-actin, showing reduced CD14 signal and diminished NF-κB nuclear accumulation after PGA1 treatment or CD14 knockdown, providing spatial validation of CD14–NF-κB pathway modulation. Scale bar = 100 μm.

To directly interrogate the roles of CD14 and PGA1 in TSPC biology, we first established a CD14-overexpressing TSPC model (CD14^hi^ TSPC) and compared four groups: NC-TSPC, CD14^hi^ TSPC, CD14^hi^ TSPC treated with free PGA1, and CD14^hi^ TSPC transfected with si-CD14. CD14 overexpression reproduced several key features observed in diabetic TSPCs: EdU incorporation was markedly reduced, cell-cycle analysis revealed G0/G1 arrest with a decreased S-phase fraction, indicating compromised proliferative capacity (Fig. 5a–d); scratch-wound assays showed impaired migratory ability (Fig. 5e); and live/dead staining together with Annexin V/PI flow cytometry demonstrated significantly increased cell death and elevated proportions of both early and late apoptotic cells (Fig. 5f–i).

On this basis, treatment with free PGA1 or si-CD14 intervention each partially reversed CD14-induced dysfunction. Both approaches increased the proportion of EdU-positive cells, restored a more physiological cell-cycle distribution, accelerated scratch closure, and reduced the percentages of dead and Annexin V/PI-positive cells (Fig. 5a–i). These findings indicate that either replenishing exogenous PGA1 or directly suppressing CD14 expression can correct CD14-mediated TSPC dysfunction at the functional level.

Mechanistically, Fig. 5 provides a systematic validation of the CD14–NF-κB–apoptosis axis. Western blotting showed that in CD14^hi^ TSPCs, pro-apoptotic proteins such as cleaved caspase-3 and Bax were upregulated, whereas the anti-apoptotic protein Bcl-2 was downregulated, indicating a shift towards a pro-apoptotic state (Fig. 5j–m). Concurrently, CD14 protein levels were markedly increased, accompanied by enhanced phosphorylation of IκBα and p65, consistent with sustained activation of the NF-κB pathway (Fig. 5n–q). Following PGA1 treatment or si-CD14 knockdown, CD14 expression was substantially reduced, levels of p-IκBα and p-p65 decreased in parallel, and these changes were accompanied by downregulation of Bax and cleaved caspase-3 together with upregulation of Bcl-2 (Fig. 5j–q), indicating effective inhibition of the CD14–NF-κB–Bax/caspase axis.

Immunofluorescence analyses further corroborated this mechanism at the spatial level: CD14 staining was markedly intensified and NF-κB showed prominent nuclear/cytoplasmic accumulation in CD14^hi^ TSPCs, whereas PGA1 or si-CD14 treatment reduced CD14 fluorescence intensity and markedly attenuated NF-κB nuclear localization (Fig. 5r and s). Taken together, the cellular and molecular evidence in Fig. 5 supports a model in which CD14 overexpression activates NF-κB signaling, enhances Bax/caspase-mediated apoptosis and suppresses Bcl-2, thereby driving TSPCs from a high-stemness state toward a “high-apoptosis, low-stemness” phenotype. Conversely, PGA1, acting as a functional ligand of CD14, and CD14 gene silencing both downregulate CD14–NF-κB signaling, thereby mechanistically explaining their protective and restorative effects on TSPC function (Fig. 5).

### 2.4 Construction, characterization and biosafety of PGA1-loaded liposomes

**Fig. 6:**
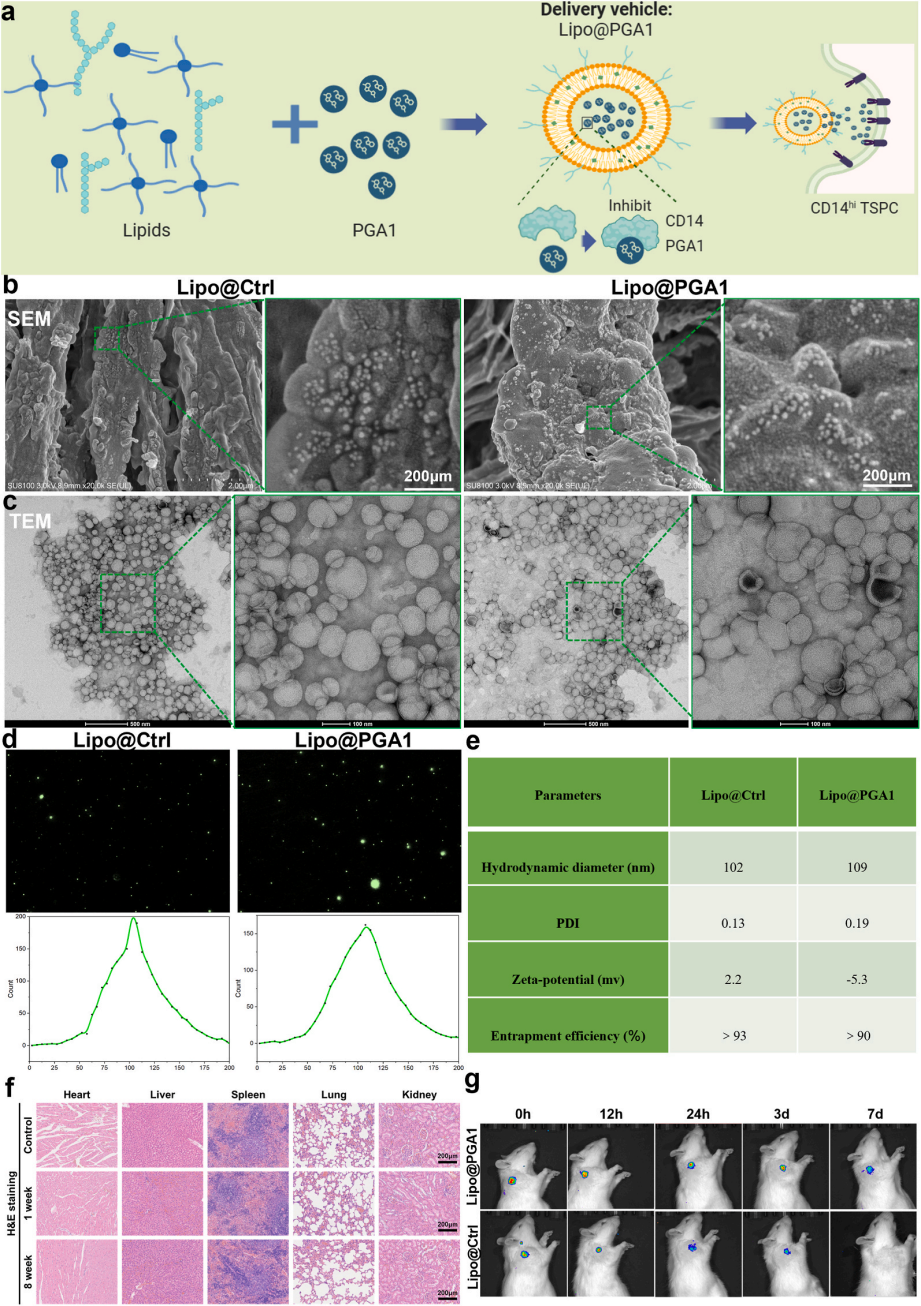
**Characterization and in vivo evaluation of PGA1-loaded liposomes (Lipo@PGA1).** (a) Schematic illustration of the preparation and delivery strategy of PGA1-loaded liposomes. PGA1 is encapsulated into a lipid bilayer vesicle (Lipo@PGA1) and delivered to CD14^hi^ TSPCs, where released PGA1 binds CD14 and inhibits CD14–NF-κB signaling. (b, c) Representative scanning electron microscopy (SEM, b) and transmission electron microscopy (TEM, c) images showing the morphology of blank liposomes and Lipo@PGA1. Both formulations display a uniform, spherical or quasi-spherical structure with smooth surfaces and a nanoscale size distribution appropriate for local tendon delivery. (d) Nanoparticle tracking analysis (NTA) of blank liposomes and Lipo@PGA1, including representative fluorescence images and size–distribution curves, confirming a narrow size distribution and good colloidal stability after PGA1 loading. (e) Physicochemical parameters of liposomal formulations. Compared with control liposomes (Lipo@Ctrl), Lipo@PGA1 shows a slightly increased hydrodynamic diameter (from 102 nm to 109 nm) and a modest change in polydispersity index (PDI; 0.13 vs. 0.19), together with a shift in zeta potential (from +2.2 mV to −5.3 mV), while maintaining high encapsulation efficiency (>93% for Lipo@Ctrl and >90% for Lipo@PGA1). (f) In vivo biocompatibility assessment of Lipo@PGA1. Representative H&E staining of major organs (heart, liver, spleen, lung and kidney) harvested after systemic or repeated local administration shows no obvious histopathological abnormalities, indicating good systemic safety of the liposomal formulation. Scale bar = 200 μm. (g) In vivo fluorescence imaging of liposomes administered around the shoulder joint of rats, demonstrating the local distribution and early retention of the liposomal signal. Lipo@PGA1 exhibited sustained accumulation in the shoulder region, supporting local retention of Lipo@PGA1 during the early postoperative period.

Given the systemic depletion of PGA1 (Fig. 2h) and its predicted binding to CD14 (Fig. 3d–n), we next engineered a PGA1-loaded liposomal formulation (Lipo@PGA1) for local delivery to diabetic tendons. In this design, PGA1 is encapsulated within a lipid bilayer vesicle that targets CD14^hi^ TSPCs at the tendon–bone interface and releases PGA1 to inhibit CD14–NF-κB signaling (Fig. 6a).

SEM and TEM imaging showed that both blank liposomes and Lipo@PGA1 were uniformly spherical or quasi-spherical nanoparticles with smooth surfaces and nanoscale dimensions suitable for local tissue penetration (Fig. 6b and c). Nanoparticle tracking analysis confirmed a narrow size distribution and good colloidal stability after PGA1 loading (Fig. 6d). Physicochemical measurements revealed that PGA1 encapsulation slightly increased the hydrodynamic diameter (102 → 109 nm) and PDI (0.13 → 0.19) and shifted the zeta potential from mildly positive to mildly negative, while maintaining high encapsulation efficiency (>90%) (Fig. 6e).

We then evaluated the safety profile of Lipo@PGA1. In vitro, human bone marrow–derived mesenchymal stem cells exposed to free PGA1, blank liposomes or Lipo@PGA1 showed no increase in DCFH-DA (DA) fluorescence (oxidative stress) and preserved BrdU incorporation, indicating that neither PGA1 nor the liposomal carrier impaired cell viability or proliferation (Supplementary Fig. 4). In vivo, repeated administration of Lipo@PGA1 in rats caused no discernible histopathological abnormalities in heart, liver, spleen, lung or kidney on Masson's trichrome staining, with preserved tissue architecture and absence of fibrosis or inflammatory infiltrates (Fig. 6f; Supplementary Fig. 7). Fluorescence imaging after peri-shoulder injection showed that the signal remained around the shoulder during the early observation period, indicating local retention at the tendon–bone interface (Fig. 6g). These data supported the use of Lipo@PGA1 as an early local delivery.

In vitro release assays further showed that Lipo@PGA1 slowed the early diffusion of PGA1. Free PGA1 diffused rapidly within 4 h, while liposomal PGA1 showed a gradual release over 72 h (Supplementary Fig. 5). In PBS (pH 7.4), PGA1 was released more slowly from Lipo@PGA1, while 10% FBS or mildly acidic acetate buffer (pH 6.5) modestly accelerated release, with cumulative release approaching 80–100% by the end of the observation period. The pH 6.5 acetate buffer was used only as an in vitro model of a mildly acidic inflammatory/wound-like environment, rather than as a measured pH value of the diabetic tendon–bone interface. Together, Lipo@PGA1 provided early controlled release and local availability of PGA1.

### 2.5 Liposomal PGA1 restores TSPC function in vitro and promotes tendon–bone healing in a diabetic RCT model

**Fig. 7:**
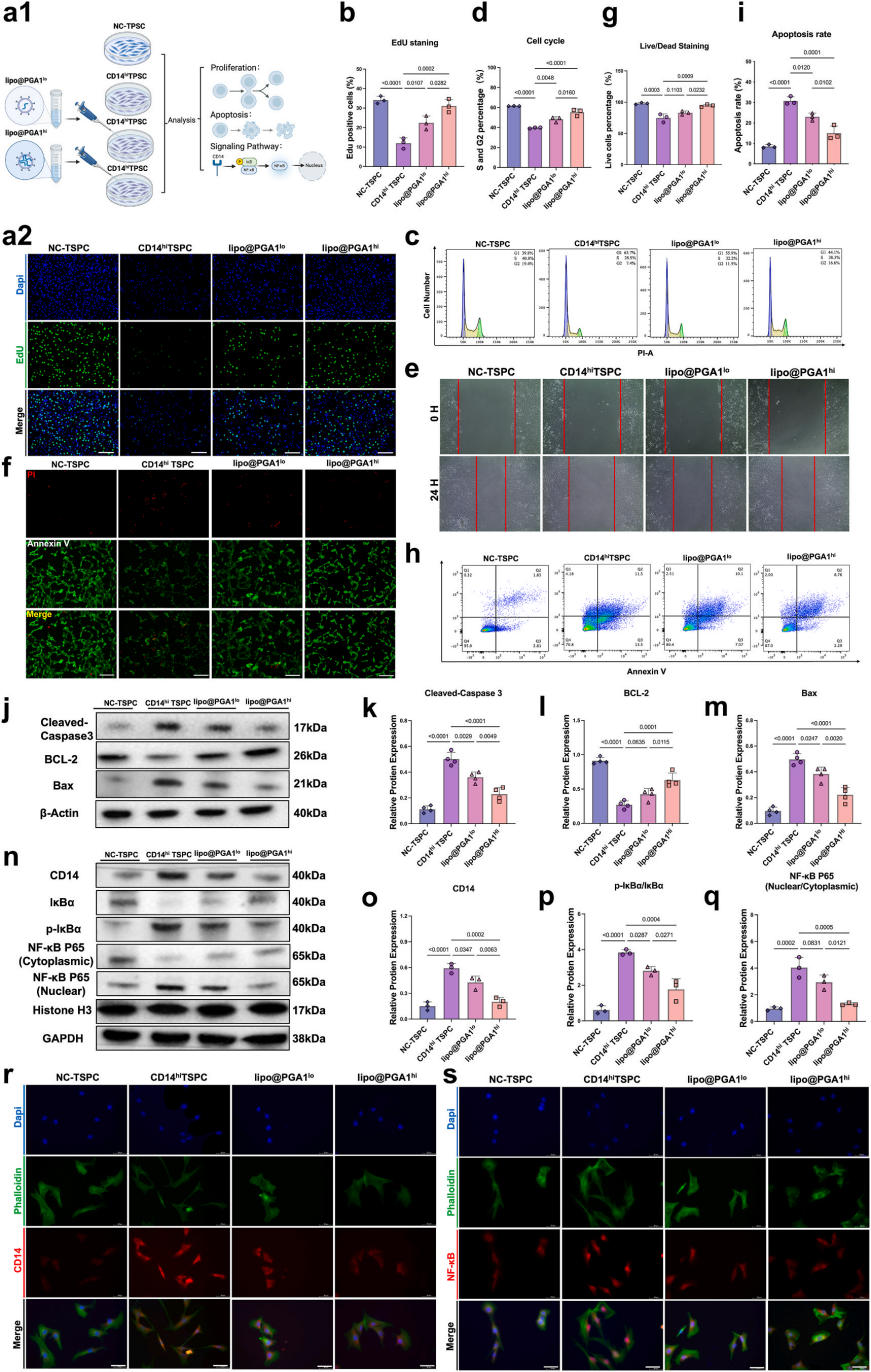
**Dose-dependent rescue of CD14-driven dysfunction in TSPCs by PGA1-loaded liposomes.** (a, b) EdU incorporation assay in TSPCs from four groups—NC-TSPC, CD14^hi^ SPC, CD14^hi^ TSPC treated with low-dose PGA1-loaded liposomes (PGA1-Lipo-Low), and CD14^hi^ treated with high-dose PGA1-loaded liposomes (PGA1-Lipo-High). Representative fluorescence images (a) and quantification of EdU-positive nuclei (b) show that CD14 overexpression suppresses proliferation, whereas PGA1-Lipo restores proliferative activity in a dose-dependent manner. Scale bar = 200 μm. (c, d) Flow-cytometric analysis of cell-cycle distribution, indicating G0/G1 arrest and reduced S-phase fraction in CD14^hi^ TSPCs, with progressive normalization of cell-cycle profiles after low- and high-dose PGA1-Lipo treatment. (e) Scratch-wound migration assay demonstrating impaired migratory capacity of CD14^hi^ TSPCs compared with NC-TSPCs, and improved wound closure following low- and high-dose PGA1-Lipo treatment. (f, g) Live/dead fluorescence staining and corresponding quantification, revealing increased dead cell proportion in CD14^hi^ TSPCs and a dose-dependent improvement in cell viability after PGA1-Lipo exposure. Scale bar = 200 μm. (h, i) Annexin V/PI flow-cytometric apoptosis analysis, with representative dot plots (h) and statistical summary of early and late apoptotic cells (i), showing enhanced apoptosis in CD14^hi^ TSPCs that is progressively attenuated by low- and high-dose PGA1-Lipo. (j–m) Western blot analysis of apoptosis-related proteins (e.g., cleaved caspase-3, Bax, Bcl-2 and other indicated markers) in the four groups, with representative blots (j–l) and densitometric quantification (m). PGA1-Lipo treatment reduces pro-apoptotic signaling and restores the pro-/anti-apoptotic balance in a concentration-dependent fashion. (n–q) Western blot analysis of CD14 and NF-κB pathway components, including CD14, phosphorylated and total IκBα, and phosphorylated and total p65, demonstrating that PGA1-Lipo downregulates CD14 expression and inhibits NF-κB activation in CD14^hi^ TSPCs, with stronger effects at the higher PGA1-Lipo dose. (r, s) Immunofluorescence staining for CD14 (r) and NF-κB (s) in TSPCs from the four groups, with DAPI-labeled nuclei and phalloidin-labeled F-actin, showing dose-dependent attenuation of CD14 signal and NF-κB nuclear accumulation following PGA1-Lipo treatment, thus providing spatial validation of CD14–NF-κB pathway inhibition by liposomal PGA1. Scale bar = 100 μm.

We next assessed whether liposomal PGA1 could rescue CD14-driven dysfunction in a dose-dependent manner. Treatment of CD14^hi^ TSPCs with low- and high-dose Lipo@PGA1 progressively increased EdU incorporation, alleviated G0/G1 arrest, and enhanced migratory capacity (Fig. 7a–e). Live/dead and Annexin V/PI assays showed a graded reduction in cell death and apoptosis with increasing PGA1 dose (Fig. 7f–i). Concordantly, pro-apoptotic protein expression and NF-κB activation were dose-dependently suppressed, as evidenced by Western blot analysis of apoptosis markers, CD14, p-IκBα and p-p65 (Fig. 7j–q). Immunofluorescence confirmed decreasing CD14 signal and NF-κB nuclear localization with escalating Lipo@PGA1 concentrations (Fig. 7r and s). Together, these data demonstrate that liposomal PGA1 can efficiently normalize CD14^hi^ TSPC behavior and signaling.

**Fig. 8:**
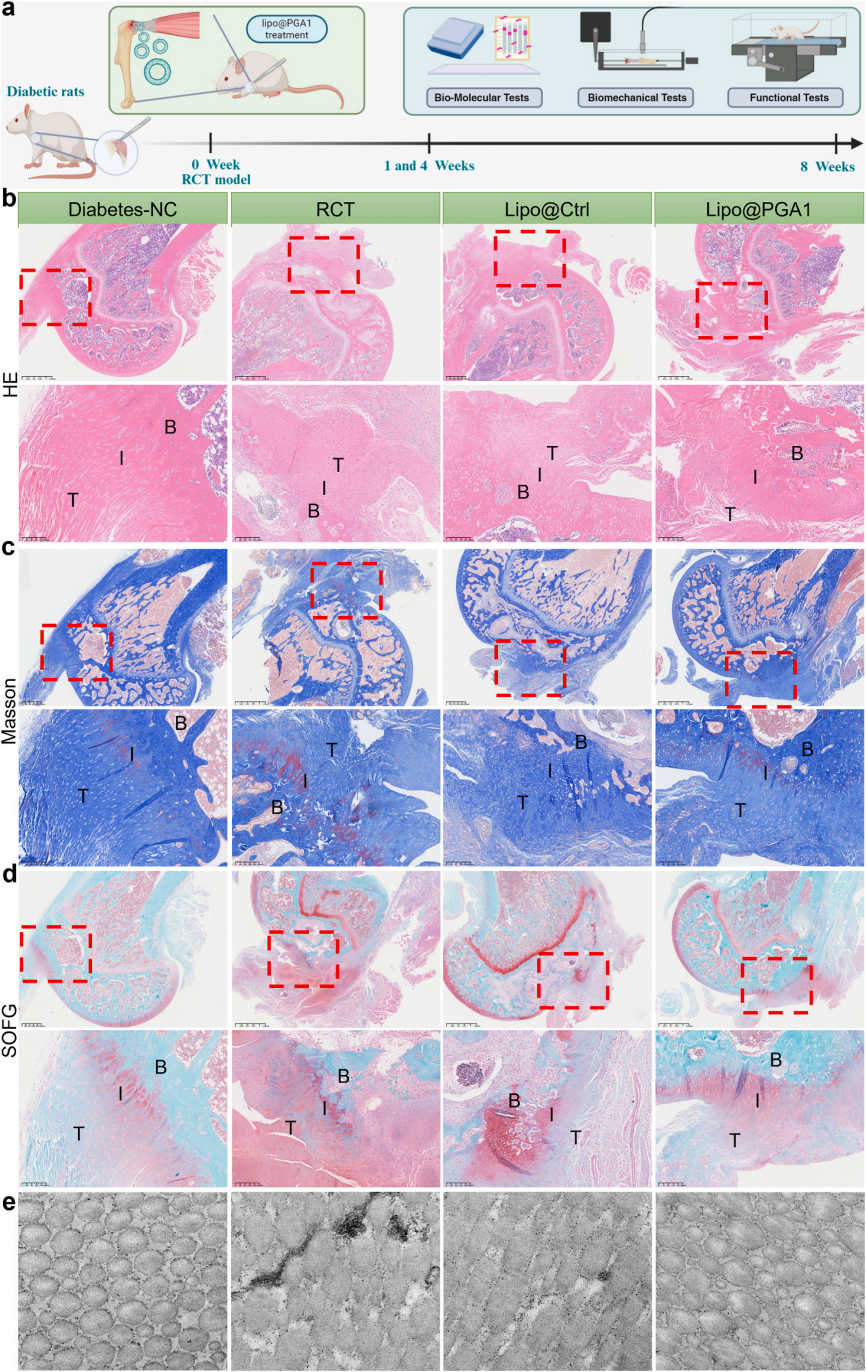
**Lipo@PGA1 improves tendon–bone healing in a diabetic rat rotator cuff tear (RCT) model.** (a) Schematic of the experimental design. Type 2 diabetic rats were subjected to supraspinatus tendon detachment and repair to establish the RCT model, followed by local administration of Lipo@PGA1 or control liposomes around the tendon–bone interface. Histological, biomechanical and functional evaluations were performed at the indicated time points. (b–d) Representative histological images of the tendon–bone interface from the four groups (Diabetes-NC, RCT, Lipo@Ctrl and Lipo@PGA1). H&E staining (b) shows improved continuity between tendon (T), interface (I) and bone (B) in the Lipo@PGA1 group, with reduced inflammatory cell infiltration and better organized repair tissue compared with untreated RCT and Lipo@Ctrl groups. Masson's trichrome staining (c) reveals denser, more regularly aligned collagen fibers and a more distinct fibrocartilaginous transition zone at the tendon–bone interface after Lipo@PGA1 treatment. Safranin O/fast green staining (d) demonstrates enhanced fibrocartilage formation and proteoglycan deposition at the insertion site in the Lipo@PGA1 group, whereas the RCT and Lipo@Ctrl groups exhibit disorganized collagen and deficient fibrocartilage. (e) Transmission electron microscopy of regenerated tendon tissue at the repair site. Tendons from Lipo@PGA1-treated rats display more uniform, tightly packed collagen fibrils with improved diameter and regular ultrastructural organization, in contrast to the loosely arranged, irregular fibrils observed in untreated RCT and Lipo@Ctrl tendons, indicating superior matrix maturation and tendon quality following Lipo@PGA1 therapy.

**Fig. 9:**
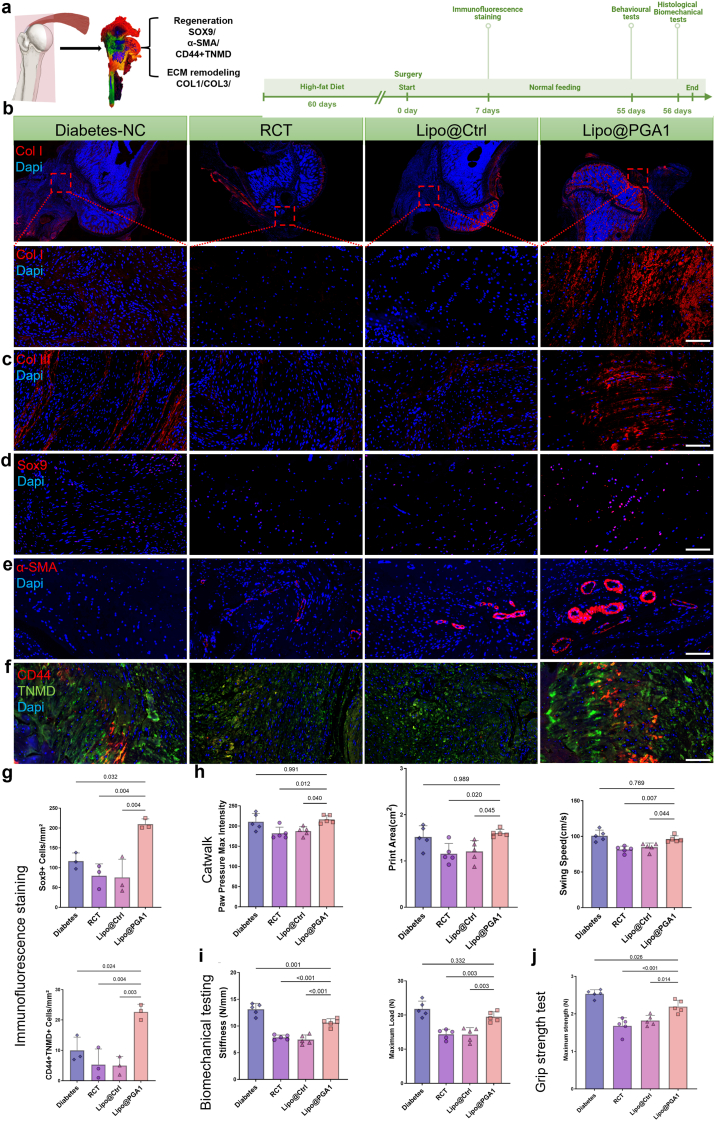
**Lipo@PGA1 enhances matrix remodeling, stemness-related markers and functional recovery in a diabetic rat RCT model.** (a) Schematic diagram of the experimental workflow in diabetic rats, including high-fat diet induction, creation and repair of the supraspinatus RCT model, local administration of Lipo@Ctrl or Lipo@PGA1, and subsequent time points for immunofluorescence, gait, biomechanical and functional assessments. (b–g) Representative immunofluorescence staining at the tendon–bone interface from the Diabetes-NC, RCT, Lipo@Ctrl and Lipo@PGA1 groups, together with semi-quantitative analysis. Scale bar = 50 μm. Collagen I (Col I) staining (b) shows restoration of dense, longitudinally aligned collagen fibers in the Lipo@PGA1 group compared with the disorganized matrix in untreated RCT and Lipo@Ctrl tendons. Collagen III (Col III) staining (c) reflects matrix remodeling at the repair interface. Sox9 staining (d) demonstrates enhanced fibrocartilage/chondrocyte-like cell presence at the insertion site after Lipo@PGA1 treatment. α-SMA staining (e) reflects myofibroblast and neovascular responses, with more organized α-SMA + structures in the Lipo@PGA1 group. Double immunofluorescence for CD44 and tenomodulin (TNMD) (f) highlights an increased population of CD44+TNMD + tendon progenitor/tenogenic cells in Lipo@PGA1-treated specimens. Bar graphs (g) summarize the semi-quantitative counts/intensities of Sox9+ cells and CD44+TNMD + cells among the four groups, confirming that Lipo@PGA1 promotes fibrocartilage regeneration and recruitment of stem/progenitor-like tenogenic cells. (h) Gait analysis performed at 8 weeks postoperatively, including paw pressure, print area and swing speed parameters of the operated forelimb. Lipo@PGA1-treated rats exhibit significantly improved paw loading and more normalized spatiotemporal gait parameters compared with RCT and Lipo@Ctrl groups, approaching those of Diabetes-NC rats. (i) Ex vivo biomechanical testing of the supraspinatus tendon–humerus complex, showing higher stiffness, maximum load to failure and ultimate stress in the Lipo@PGA1 group relative to untreated RCT and Lipo@Ctrl tendons, indicating superior structural integrity of the repaired insertion. (j) In vivo forelimb grip strength test demonstrating that Lipo@PGA1 significantly enhances functional strength of the operated limb compared with RCT and Lipo@Ctrl groups, consistent with the histological and biomechanical improvements at the tendon–bone interface.

To translate these findings in vivo, we established a diabetic rat supraspinatus rotator cuff tear (RCT) model (Supplementary Fig. 6) and administered Lipo@PGA1 or control liposomes around the tendon–bone interface (Figs. 8a and 9a). Histological analyses at follow-up revealed that untreated RCT tendons in diabetic rats exhibited disrupted tendon–bone continuity, marked inflammatory infiltration, loose collagen arrangement and deficient fibrocartilage, whereas Lipo@PGA1-treated tendons showed well-integrated tendon–bone interfaces, reduced inflammation, denser and more orderly collagen fibers, and robust fibrocartilage and proteoglycan deposition (Fig. 8b–d). Ultrastructurally, TEM demonstrated that Lipo@PGA1 promoted the formation of uniformly packed collagen fibrils with regular diameter, in contrast to the irregular, loosely arranged fibrils observed in RCT and Lipo@Ctrl groups (Fig. 8e). Semi-quantitative histological scoring confirmed significant improvement in tendon–bone healing in the Lipo@PGA1 group compared with RCT and Lipo@Ctrl groups (Supplementary Fig. 8).

At the molecular level, immunofluorescence staining showed that Lipo@PGA1 was associated with improved collagen remodeling, characterized by enhanced collagen I organization and a more balanced collagen I/III matrix pattern, together with increased Sox9+ fibrocartilage-like cells and more organized α-SMA + vascular/perivascular structures (Fig. 9b–e). Moreover, Lipo@PGA1 increased the abundance of CD44+TNMD + tendon progenitor/tenogenic cells, indicating improved recruitment or maintenance of a regenerative TSPC pool (Fig. 9f and g). Western blotting of insertion tissues revealed that CD14 expression, markedly elevated after RCT, was partially reduced by control liposomes and most strongly downregulated by Lipo@PGA1 at the tendon-bone interface (Supplementary Fig. 9), consistent with the proposed mechanism of CD14 targeting.

Functionally, gait analysis demonstrated that Lipo@PGA1-treated rats exhibited significantly improved paw pressure, print area and swing speed of the operated limb compared with RCT and Lipo@Ctrl groups, approaching values seen in Diabetes-NC rats (Fig. 9h). Biomechanical testing of the supraspinatus tendon–humerus complex showed higher stiffness, maximum load to failure and ultimate stress in the Lipo@PGA1 group, indicating superior mechanical integrity of the repaired insertion (Fig. 9i). In vivo forelimb grip-strength measurements further confirmed that Lipo@PGA1 enhanced functional strength of the operated limb relative to untreated RCT and Lipo@Ctrl rats (Fig. 9j). Collectively, these data establish that targeted delivery of PGA1 via Lipo@PGA1 effectively improves tendon–bone healing and shoulder function in diabetic RCT, without inducing systemic toxicity (Fig. 6f; Supplementary Fig. 7).

**Fig. 10:**
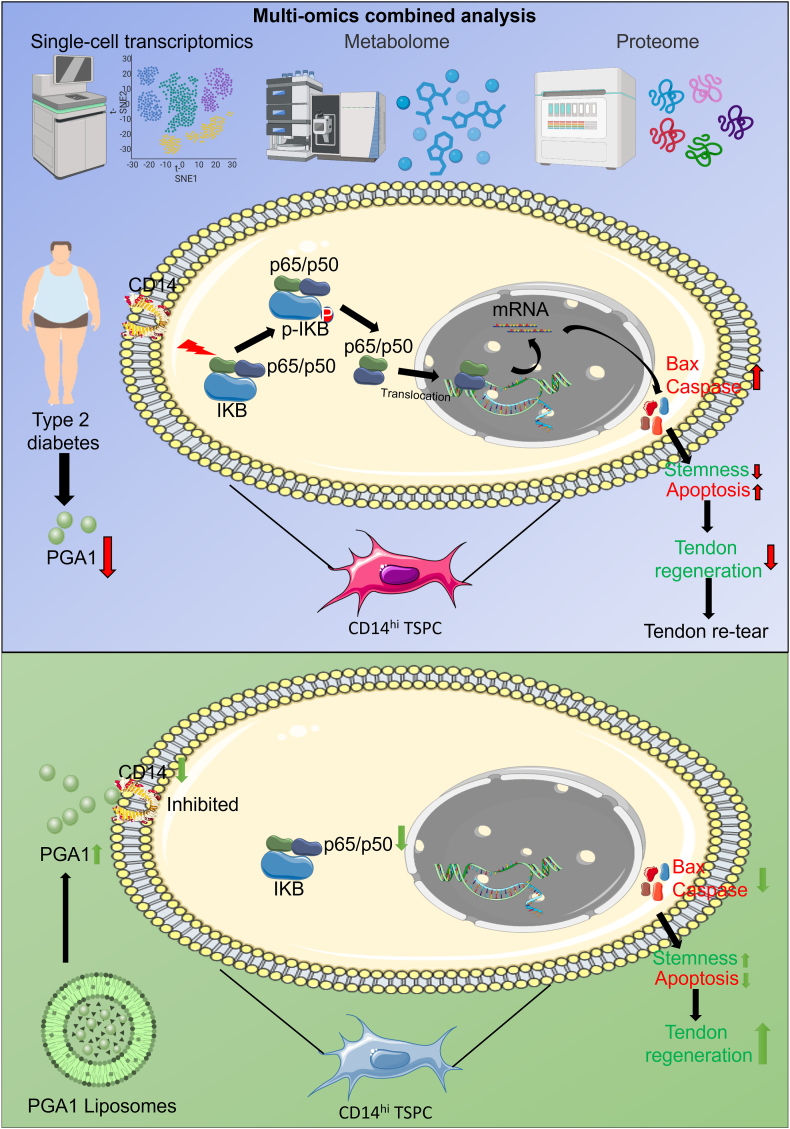
**Schematic summary of the multi-omics–guided mechanism by which PGA1-loaded liposomes restore TSPC function and promote tendon regeneration in type 2 diabetes.** Single-cell transcriptomics, serum metabolomics and tendon proteomics jointly reveal that in type 2 diabetes circulating PGA1 levels are reduced and tendon-derived TSPCs exhibit CD14 upregulation with activation of the NF-κB pathway. In CD14^hi^ TSPCs (upper panel), CD14-mediated signaling promotes IκB phosphorylation and degradation, nuclear translocation of p65/p50, and transcription of pro-apoptotic genes such as Bax and caspases, leading to increased apoptosis, loss of stemness, impaired tendon regeneration and a higher risk of tendon re-tear. Local delivery of PGA1 via a PGA1-loaded injectable liposomes (Lipo@PGA1, lower panel) replenishes PGA1 in the diabetic tendon microenvironment and directly inhibits CD14. This suppresses NF-κB activation, reduces Bax/caspase-mediated apoptosis, preserves TSPC stemness, and ultimately enhances tendon regeneration and structural integration at the tendon–bone interface.

In summary, integrating the multi-omics, cellular, biomaterial and in vivo data, we propose a model in which type 2 diabetes reduces systemic PGA1 levels and induces CD14 upregulation in tendon-resident TSPCs, leading to NF-κB activation, increased Bax/caspase-dependent apoptosis, loss of stemness and impaired tendon regeneration (Fig. 1, Fig. 2, Fig. 3, Fig. 4, Fig. 10). Early local delivery of PGA1 using Lipo@PGA1 was associated with reduced CD14 expression at the repair interface and suppression of CD14–NF-κB signaling in CD14^hi^ TSPCs, thereby improving proliferative, migratory and survival capacities (Fig. 5, Fig. 7). In a clinically relevant diabetic RCT model, this targeted strategy enhances matrix remodeling, fibrocartilage formation, recruitment of CD44+TNMD + progenitor cells and ultimately improves tendon–bone integration, biomechanical strength and limb function (Fig. 8, Fig. 9), while maintaining favorable local and systemic safety profiles (Fig. 6). This work identifies the CD14–PGA1 axis as a critical regulator of TSPC fate in diabetic tendon and provides a mechanistically informed biomaterial-based approach to promote tendon regeneration under metabolic disease conditions.

## 3 Discussion

In this study, we investigated how T2DM perturbs TSPC biology and tendon–bone healing, and we developed a multi-omics–guided, biomaterial-based strategy using PGA1-loaded liposomes to restore CD14^hi^ TSPCs function. By integrating tendon single-cell RNA sequencing, serum metabolomics and tendon proteomics with mechanistic in vitro assays and a diabetic RCT model, we identify a CD14–NF-κB inflammatory axis, coupled to systemic depletion of the pro-resolving lipid mediator PGA1, as a central mechanism linking diabetes to TSPC apoptosis, loss of stemness and impaired tendon–bone integration. Local delivery of PGA1 via a slow-release liposomal formulation restored TSPC stemness, improved matrix remodeling and fibrocartilage formation at the tendon–bone interface, and significantly enhanced biomechanical and functional outcomes in diabetic RCT, without evident systemic toxicity.

T2DM is increasingly recognized as a major comorbidity in patients with RCT and is associated with poorer clinical and structural outcomes after rotator cuff repair [6,7,9]. Previous clinical and epidemiologic studies have shown that diabetic patients exhibit higher incidence of RCT, higher retear rates and less favorable functional recovery than non-diabetic individuals, even after adjustment for age and tear size [8,10,11]. Hyperglycemia, advanced glycation end-product (AGE) accumulation, microangiopathy and chronic low-grade inflammation contribute to a hostile tendon microenvironment characterized by impaired collagen cross-linking, disorganized ECM and reduced regenerative capacity [12,13,15,16]. Similar to osteoporosis, where low bone mineral density and altered bone remodeling compromise anchor fixation and tendon–bone integration, diabetes appears to act as an independent biological stressor on tendon and enthesis tissues, rather than merely reflecting age or mechanical overload [13,14]. However, unlike osteoporosis—where antiresorptive drugs or bone-strengthening strategies can be incorporated into perioperative management—there are currently no targeted therapies that specifically correct the diabetic tendon microenvironment or restore diabetic TSPC function [6,7,11]. Our data addresses this unmet need by defining a diabetes-specific molecular axis in TSPCs and demonstrating a feasible local intervention to modulate it. Because the present study focused on isolating the local effect of Lipo@PGA1 on the diabetic tendon microenvironment, systemic antidiabetic drugs such as metformin were not combined with the treatment. Glycemic control may influence inflammatory burden, CD14 activation and repair capacity, and future studies should evaluate Lipo@PGA1 under different glycemic-control conditions and in combination with standard antidiabetic therapies. Our findings extend this concept by suggesting that diabetes impairs tendon–bone healing not only through matrix and vascular abnormalities, but also through dysfunction of tendon-resident progenitor-like cells.

TSPCs are increasingly appreciated as key orchestrators of tendon homeostasis and tendon–bone healing [25,38]. Consistent with previous work, our scRNA-seq analyses confirmed the presence of a distinct TSPC compartment within tendon tissue and revealed that T2DM profoundly remodels its cellular composition and transcriptional states. Diabetic tendons displayed a redistribution of tendon-resident cell populations with prominent alteration of TSPC subclusters, including enrichment of inflammatory and stress-related transcriptional programs, reduced expression of stemness-associated genes and perturbation of ECM-organization pathways. Functionally, diabetic TSPCs exhibited reduced proliferative capacity, defective cell-cycle progression, impaired migration and increased apoptosis, together indicating a shift toward a low-stemness, pro-degenerative state. These findings extend prior observations that age-, osteoporosis- or injury-related changes can drive tendon progenitors toward senescence-like or fibroblastic phenotypes with diminished regenerative potential, and they suggest that metabolic disease adds a further layer of dysfunction at the level of resident tendon stem cells [[39], [40], [41]].

A key mechanistic insight of this work is the identification of CD14–NF-κB signaling as a central node in diabetic TSPC dysfunction. Our multi-omics analysis showed robust upregulation of CD14 at the transcript and protein level in TSPCs derived from diabetic tendons, along with enrichment of NF-κB and other inflammatory pathways. Functional assays confirmed that CD14 CD14^hi^ TSPCs recapitulate the diabetic phenotype, including impaired proliferation and migration, G0/G1 arrest, and Bax/caspase-3–mediated apoptosis with reduced Bcl-2 expression. At the signaling level, CD14 overexpression was associated with increased phosphorylation of IκBα and p65 and nuclear accumulation of NF-κB, supporting sustained pathway activation. These data are consistent with the established role of CD14 as a co-receptor for damage-associated molecular patterns (DAMPs) and pathogen-associated molecular patterns (PAMPs), amplifying inflammatory signaling in myeloid and stromal cells via NF-κB activation [42,43]. Consistent with the phosphorylation data, immunofluorescence analysis showed that PGA1 treatment or CD14 knockdown reduced NF-κB p65 nuclear accumulation in CD14^hi^ TSPCs, indicating inhibition of NF-κB activation and nuclear translocation. Other diabetes-related pathways identified by the omics analyses, including insulin/IGF, AMPK/mTOR, oxidative stress and ECM-remodeling pathways, may also interact with CD14–NF-κB signaling and contribute to diabetic tendon degeneration. In the diabetic CD14 tendon niche, where AGEs, lipotoxic intermediates and low-grade inflammation are prevalent, heightened CD14 expression on TSPCs likely sensitizes these cells to inflammatory stimuli, driving them toward apoptosis and stemness exhaustion rather than regeneration [43,44].

It should be noted that the CD14^hi^ TSPC model was used as a reductionist tool to examine the CD14-dependent component of diabetic TSPC dysfunction. This model does not fully reproduce the complex diabetic microenvironment. Nevertheless, the relevance of this model was supported by primary diabetic TSPCs, which showed similar functional impairment and activation of CD14–NF-κB signaling. The metabolomic analysis also pointed to insulin/IGF, AMPK, mTOR and FOXO-related pathways, which are closely related to systemic diabetic metabolism. These pathways shaped the overall tendon microenvironment, but they are broad metabolic networks and were not the main targets of the present local intervention. We focused on CD14–NF-κB signaling because it was altered in the tendon progenitor-like compartment and matched the cellular phenotype observed in this study, including apoptosis, cell-cycle arrest and impaired migration. This provided a more direct link between PGA1 depletion, CD14 activation and TSPC dysfunction.

Our multi-omics platform further linked these cellular changes to systemic metabolic remodeling in diabetes. Serum metabolomics linked diabetes to broad lipid-metabolic remodeling, among which the reduction of PGA1 was particularly relevant to the inflammatory phenotype observed in diabetic TSPCs. PGA1 and related specialized pro-resolving mediators are known to dampen NF-κB activation, promote resolution of inflammation and support tissue repair in diverse organs [45,46]. Here, the combination of multi-omics profiling and molecular docking/molecular dynamics simulations suggested that CD14 may act as a putative PGA1-associated receptor involved in inflammatory signaling regulation in TSPCs. Together with the functional rescue data, these findings suggested that PGA1 depletion may weaken an endogenous restraint on CD14-associated NF-κB signaling, thereby promoting apoptosis and reduced progenitor-like function in diabetic TSPCs.

The functional rescue experiments with free PGA1 and si-CD14 provide further mechanistic validation of this PGA1–CD14–NF-κB axis. In CD14^hi^ TSPCs, both PGA1 supplementation and CD14 knockdown improved proliferation, migration and survival. At the molecular level, these interventions suppressed NF-κB activation and reduced Bax/caspase-related apoptotic signaling. Importantly, immunofluorescence revealed decreased nuclear NF-κB accumulation under both treatments, reinforcing the concept that PGA1 acts as a functional ligand that restrains CD14-driven NF-κB activation. Together, these data support a model in which the CD14–PGA1 axis operates as a central rheostat of TSPC fate in diabetes: when PGA1 is depleted, CD14 signaling predominates and drives TSPCs toward a “high-apoptosis, low-stemness” phenotype; when PGA1 is replenished or CD14 is silenced, NF-κB activity is curtailed, apoptosis decreases and TSPC stemness is restored.

From a translational perspective, local delivery of small lipophilic mediators such as PGA1 poses formulation and pharmacokinetic challenges. Systemic administration risks off-target effects and rapid clearance, while simple local injection may not sustain effective concentrations at the tendon–bone interface over the prolonged healing period [47,48]. To address this, we engineered PGA1-loaded liposomes as a local early-release system. The liposomal formulation provided a biocompatible carrier for local PGA1 delivery, with physicochemical properties suitable for in vivo administration. Importantly, in vivo imaging demonstrated that peri-shoulder injection resulted in localized and sustained retention of liposomes around the tendon–bone interface, supporting their suitability as a depot for PGA1 delivery. Although this study focused on CD14^hi^ TSPCs, the in vivo effects of Lipo@PGA1 might also involve indirect regulation of macrophage activity, fibroblast remodeling and matrix organization through changes in the local progenitor-cell microenvironment. Future co-culture or spatially resolved analyses will help clarify these cell–cell interactions.

Additionally, the present retention and release data do not define the in vivo metabolic fate, effective PGA1 concentration window or optimal injection interval of Lipo@PGA1. Future dose-optimization studies should combine quantitative PGA1 measurement in tendon–bone interface tissues, plasma pharmacokinetic analysis, biodistribution assessment and repeated-dose safety evaluation to guide clinically relevant administration regimens. The improvements observed at 4 and 8 weeks were therefore more likely to result from early modulation of the diabetic repair microenvironment. Early inhibition of CD14–NF-κB signaling might reduce progenitor-cell apoptosis, preserve a more regenerative tendon progenitor-like cell pool, and shift the repair process toward more organized matrix remodeling. These early changes subsequently led to improved fibrocartilage formation, collagen organization and mechanical strength at later stages of tendon–bone healing. Although the 8-week endpoint is commonly used to assess tendon–bone healing in rodent rotator cuff repair models, these data do not establish long-term repair durability or retear prevention. Longer follow-up studies at 12 or 24 weeks will be needed to determine whether Lipo@PGA1 improves late-stage remodeling stability.

The liposomal formulation used here was intentionally kept simple and injectable. The goal of this study was not to provide mechanical reinforcement of the tendon–bone interface, but to test whether local delivery of PGA1 could modulate the CD14–NF-κB inflammatory-apoptotic axis in diabetic tendon progenitor-like cells. More complex scaffolds or hydrogels may provide mechanical or topographical cues, but they could also make it more difficult to determine whether the observed biological effects were caused by PGA1 itself or by the carrier structure. Nevertheless, the tendon–bone interface is mechanically demanding, and a liposomal suspension does not provide structural reinforcement. Future studies may integrate Lipo@PGA1 with hydrogels, electrospun scaffolds or other mechanically instructive biomaterials to improve retention and mechanical compatibility in large defects, chronic tears or revision repair settings.

The use of mice and rats reflects the different technical requirements of the study. Mice were used for the omics and in vitro experiments because they were well suited for tendon scRNA-seq, molecular profiling and TSPC isolation. The rat supraspinatus repair model was then used for in vivo validation, as its larger tendon–bone interface permited more reliable surgery, local administration and mechanical testing. This strategy allowed us to identify the CD14–PGA1–NF-κB axis in mouse tendon/TSPCs and test whether targeting this axis could improve tendon–bone healing in a surgically relevant rat model.

Functionally, Lipo@PGA1 preserved the protective effect of PGA1 in CD14 CD14^hi^ TSPCs. Its effects on proliferation, migration, survival and NF-κB activation were consistent with the proposed CD14-dependent mechanism. These data indicate that liposomal encapsulation not only enables local delivery but also maintains sufficient exposure to modulate TSPC signaling over time.

In the diabetic rat supraspinatus RCT model, Lipo@PGA1 translated into robust structural and functional benefits. The histological and ultrastructural findings indicated more organized matrix remodeling and fibrocartilage formation. At the molecular level, the collagen I/III findings demonstrated improved collagen balance and organization. Collagen III can participate in active repair, and α-SMA may mark either fibrotic myofibroblast activation or organized vascular/perivascular repair structures depending on its spatial pattern. In the Lipo@PGA1 group, these signals were associated with improved collagen organization and fibrocartilage formation, supporting a more coordinated repair response rather than pathological fibrosis. Notably, CD14 expression at the tendon–bone interface was reduced after Lipo@PGA1 treatment, which was consistent with the proposed CD14–PGA1–NF-κB mechanism.

These structural and molecular changes translated into meaningful functional gains. Gait analysis showed improved paw loading and more normalized spatiotemporal parameters in Lipo@PGA1-treated animals, approaching values of non-operated diabetic controls. Biomechanical testing demonstrated higher stiffness, maximum load to failure and ultimate stress of the supraspinatus tendon–humerus complex, indicating superior mechanical integrity of the repaired insertion. In vivo forelimb grip strength was likewise improved in the Lipo@PGA1 group, confirming better functional recovery of the operated limb. Taken together, our findings suggest that multi-omics–guided targeting of the CD14–NF-κB axis with PGA1-loaded liposomes effectively reprograms the diabetic tendon niche, restores CD14^hi^ TSPCs function and improves tendon–bone healing in the context of metabolic disease.

## 4 Limitations

Several limitations should be considered. First, the multi-omics discovery was performed mainly in mouse Achilles tendon, whereas therapeutic validation was conducted in a rat supraspinatus repair model; therefore, species- and tendon-site differences may affect direct extrapolation. Second, although both mouse and rat T2DM models were used, rodent models cannot fully reproduce the long disease duration, glycemic variability, medication exposure and systemic complications of human diabetes. Third, the diabetic models were diet- and/or pharmacologically induced, and whether the PGA1–CD14–NF-κB alterations occur across different etiologies and severities of human T2DM remains unclear. Fourth, this study focused mainly on TSPCs and the tendon–bone interface, while other diabetes-relevant cell types, such as macrophages, endothelial cells and pericytes, were not mechanistically dissected. Fifth, although short-to mid-term safety of Lipo@PGA1 was evaluated, its long-term toxicity, optimal dosing and interactions with systemic antidiabetic therapies remain to be defined. Sixth, the scRNA-seq-defined TSPC population may include tendon fibroblast-like cells with progenitor features; thus, TSPCs are used here as an operational term for a TSPC-enriched progenitor-like stromal population. Seventh, molecular docking and MD simulations predicted a stable PGA1–CD14 interaction, but direct biochemical binding was not validated. Eighth, CD14/TSPC-marker co-localization staining at the tendon–bone interface was not performed, limiting cell-type-specific spatial interpretation of CD14 modulation in vivo. Ninth, the in vitro rescue experiments mainly used CD14^hi^ TSPCs, and more complex high-glucose-, palmitate- or AGE-treated models are needed to better mimic the diabetic microenvironment. Tenth, human serum and rotator cuff tissue validation was not included, so PGA1 depletion and CD14 upregulation in human diabetic rotator cuff disease remain to be confirmed. Finally, the mechanistic analysis centered on the CD14–NF-κB–Bax/caspase axis, while additional pathways related to immunometabolism, oxidative stress and matrix remodeling may also contribute to diabetic tendon pathology.

## 5 Conclusion

In conclusion, this study identifies a PGA1–CD14–NF-κB axis as a critical regulator of TSPC fate and tendon–bone healing in the setting of T2DM. Multi-omics profiling revealed that diabetic tendons harbor CD14^hi^, low-stemness TSPCs within an inflammatory, degenerative microenvironment, while systemic PGA1 levels are markedly reduced. Mechanistic assays demonstrated that CD14 overexpression drives NF-κB–dependent apoptosis and loss of stemness in TSPCs, whereas PGA1 supplementation or CD14 silencing reverses these defects. Guided by these insights, we engineered PGA1-loaded liposomes that safely deliver PGA1 to the tendon–bone interface, suppress CD14–NF-κB signaling, restore CD14^hi^ TSPCs function and significantly enhance tendon–bone integration, matrix quality, biomechanics and shoulder function in a diabetic RCT model. These findings not only deepen our understanding of how metabolic disease impairs tendon regeneration, but also propose multi-omics–guided, CD14–NF-κB–targeting liposomal PGA1 as a promising therapeutic strategy to improve tendon–bone healing in diabetic patients.

### 5.1 Materials and methods

Please find the details in the supplemental files.

## Funds

This work was supported by the Suzhou Clinical Key Disease Diagnosis and Treatment Technology Special Project (LCZX202127), the 10.13039/100018696Kunshan High-Level Health Talent Project (Kunwei [2024] No. 9), Key Supported Discipline Construction Project of Suzhou (SZFCXK202115), and the Golden Apricot Superior Talent Project of Kunshan Hospital of Traditional Chinese Medicine (03rczc25).

## CRediT authorship contribution statement

**Minghao Tong:** Data curation, Formal analysis, Investigation. **Liyan Liu:** Data curation, Formal analysis, Investigation. **Wanshun Liu:** Data curation, Formal analysis, Investigation. **Haocheng Qin:** Data curation, Formal analysis, Investigation. **Zhixuan Mai:** Investigation. **Pingkang Qian:** Investigation. **Yinhua Qian:** Investigation. **Haoqiang Huang:** Investigation. **Quan Yang:** Investigation. **Xiaofeng Wu:** Investigation. **Chen Kuang:** Investigation. **Yanwei He:** Investigation. **Renwen Wan:** Investigation. **Wei Luo:** Investigation. **Yuxi Ou:** Investigation. **Feng Xu:** Conceptualization, Funding acquisition, Investigation. **Xiaolan Cheng:** Conceptualization, Funding acquisition, Investigation. **Zhiwen Luo:** Conceptualization, Funding acquisition, Investigation. **Qing Wang:** Conceptualization, Funding acquisition, Investigation.

## Declaration of competing interest

The authors declare that they have no known competing financial interests or personal relationships that could have appeared to influence the work reported in this paper.

## Data Availability

Data will be made available on request.
