# Supplementary material for: Multi-omics-guided targeting of the CD14–NF-κB axis with PGA1-loaded liposomes restores CD14hi TSPCs function and promotes tendon–bone healing in diabetes
Source: Mater Today Bio. 2026 Jul 30;40:103509. doi: 10.1016/j.mtbio.2026.103509 (PMC13476539; doi:10.1016/j.mtbio.2026.103509)
Supplement: Supplementary file 1 [file mmc1.docx]

Supplementary Materials for

**Multi-omics-guided targeting of the CD14–NF-κB axis with PGA1-loaded liposomes restores CD14^hi^ TSPCs function and promotes tendon–bone healing in diabetes**

**Authors:** *Minghao Tong^1,#^, Liyan Liu^2,#^, Wanshun Liu^3,#^, Haocheng Qin^4,#^, Zhixuan Mai^5^, Pingkang Qian^1^, Yinhua Qian^1^, Haoqiang Huang^1^, Quan Yang^1^, Xiaofeng Wu^1^, Chen Kuang^1^, Yanwei He^5^, Renwen Wan^5^, Wei Luo^5^, Yuxi Ou^6^, Feng Xu^1,*^, Xiaolan Cheng^7,*^, Zhiwen Luo^3,5,8, *^, Qing Wang^1,*^*

Corresponding author: doctorwg1983@njmu.edu.cn, [zhiwen.luo_fudan@hotmail.com](mailto:zhiwen.luo_fudan@hotmail.com), [chengxiaolan37@njucm.edu.cn](mailto:chengxiaolan37@njucm.edu.cn), [xf701228@163.com](mailto:xf701228@163.com)

**The PDF file includes:**

Materials and Methods

Figs. S1 to S9

References

**Other Supplementary Materials for this manuscript include the following:**

**Materials and Methods**

**1. Animal models and sample collection**

**1.1 Ethics approval and animal husbandry**

This work involving animal subjects has been reviewed and approved by the Animal Ethics Committee (AEC) of Leadgene Biotechnology (Kunshan) Co., Ltd. All experimental procedures were conducted in strict compliance with the committee's guidelines, which are based on internationally accepted principles for the ethical use and care of laboratory animals, including the "3Rs" (Replacement, Reduction, and Refinement). The approved protocol number is 20241024-0076-01. Male C57BL/6J mice (8–10 weeks, 22–25 g) and male Sprague–Dawley rats (8–10 weeks, 250–300 g) were obtained from Shanghai SLAC Laboratory Animal Co., Ltd. (Shanghai, China). Animals were housed in specific pathogen–free (SPF) facilities under controlled temperature (22 ± 2 °C), humidity (50 ± 10%) and a 12 h light/dark cycle with free access to standard chow and water, unless otherwise specified. Animals were acclimatized for at least 1 week before experiments.

**1.2 Induction of type 2 diabetes mellitus in mice and rats**

Type 2 diabetes mellitus (T2DM) was induced using a high-fat diet (HFD) combined with low-dose streptozotocin (STZ) as previously described with minor modifications. Briefly, mice and rats in the diabetes groups were fed HFD (60% kcal from fat, Shanghai SLAC Laboratory Animal Co., Ltd., Shanghai, China) for 4 weeks. Thereafter, animals received intraperitoneal injections of STZ (35 mg/kg for mice, 30 mg/kg for rats; Sigma–Aldrich, USA) freshly dissolved in 0.1 M citrate buffer (pH 4.5) for 5 consecutive days^1^. Control animals received vehicle injections and were maintained on standard chow.

Fasting blood glucose (FBG) was measured weekly from tail vein blood using a glucometer (Accu-Chek, Roche, Switzerland) after 12 h fasting. Animals with FBG ≥ 11.1 mmol/L on two consecutive measurements and exhibiting polyphagia/polydipsia and weight gain compared with baseline were considered diabetic and used for subsequent experiments. Body weight and FBG curves are shown in Supplementary Fig. 1 and Supplementary Fig. 6.

**1.3 Mouse tendon and serum collection for multi-omics analyses**

Three months after successful induction of T2DM, tendons and blood were collected from diabetic (DM_T) and age-matched normoglycemic control (NC_T) mice. Under deep anesthesia with pentobarbital sodium (50 mg/kg, i.p.), whole blood was collected via cardiac puncture, allowed to clot at room temperature, and centrifuged at 3,000 × g for 10 min to obtain serum, which was stored at −80 °C for metabolomic analysis. Achilles tendons were carefully dissected under sterile conditions. Portions were used fresh for single-cell dissociation; the remaining tissue was snap-frozen in liquid nitrogen and stored at −80 °C for proteomic analysis.

**2. Multi-omics analysis**

**2.1 Single-cell suspensions, library construction and single-cell RNA sequencing**

Fresh Achilles tendons from NC_T and DM_T mice (n = 3 per group) were minced into 1–2 mm³ fragments in ice-cold PBS and digested in an enzyme cocktail containing collagenase type I (2 mg/mL, Worthington, USA), dispase II (2 mg/mL, Roche, Switzerland) and DNase I (50 U/mL, Sigma–Aldrich) in DMEM at 37 °C for 1.5–2 h with gentle agitation^2^. Digestion was terminated by adding an equal volume of DMEM containing 10% fetal bovine serum (FBS, Gibco, USA). The cell suspension was filtered through a 70 µm cell strainer, centrifuged at 300 × g for 5 min, and resuspended in 0.04% BSA/PBS. Cell viability (>85%) was assessed by trypan blue exclusion.

Single-cell libraries were prepared using the Chromium Single Cell 3’ v3.1 kit and Chromium Controller (10x Genomics, USA) following the manufacturer’s protocol. Briefly, 8,000–10,000 cells per sample were loaded to generate gel bead-in-emulsions (GEMs), followed by reverse transcription, cDNA amplification, library construction and index PCR. Libraries were quantified by Qubit (Thermo Fisher Scientific, USA) and Bioanalyzer (Agilent, USA) and sequenced on an Illumina NovaSeq 6000 platform to achieve a depth of ~50,000 reads per cell.

**2.2 scRNA-seq data processing, clustering and enrichment analysis**

Raw sequencing data were processed using Cell Ranger (v6.1, 10x Genomics) to demultiplex reads, align them to the mm10 mouse reference genome, and generate gene–barcode matrices. Downstream analysis was performed in R (v4.2.0) using Seurat (v4.3.0). Cells with 200–6,000 detected genes, 500–100,000 UMIs and <25–30% mitochondrial gene content were retained. Doublets were filtered using the DoubletFinder package^3,4^.

Gene expression data were normalized (“NormalizeData”), scaled (“ScaleData”), and the top 2,000 highly variable genes were selected for principal component analysis (PCA). The first 20–30 principal components were used for graph-based clustering (“FindNeighbors” and “FindClusters”, resolution 0.8–1.2) and visualization by UMAP. Initial transcriptomic clusters were generated by unsupervised graph-based clustering and visualized by UMAP. Cell-type annotation was then performed according to canonical tendon and stromal marker genes together with literature-based tendon cell signatures. Representative markers used for cluster identification are shown in Supplementary Fig. S3. Because tendon fibroblastic and progenitor-like cells form a transcriptional continuum, the population originally annotated as TSPCs is referred to as TSPC-enriched progenitor-like stromal cells where appropriate.

TSPCs were subsetted and reclustered to define transcriptionally distinct subpopulations. Differentially expressed genes (DEGs) between NC_T and DM_T TSPCs were obtained using Wilcoxon rank-sum tests (“FindMarkers”, |log2FC| ≥ 0.25, adjusted P < 0.05). Gene Ontology (GO), KEGG pathway enrichment and gene set enrichment analysis (GSEA) of DEGs were performed using the clusterProfiler package. NF-κB- and inflammation-related signatures, stemness-associated signatures and ECM homeostasis signatures were scored at the single-cell level using the AUCell method.

**2.3 Serum untargeted metabolomics and pathway analysis**

Serum samples from NC and diabetic mice (n = 6 per group) were thawed on ice. For extraction, 50–100 µL serum was mixed with 3–4 volumes of ice-cold methanol/acetonitrile (1:1, v/v), vortexed for 60 s and incubated at −20 °C for 1 h to precipitate proteins. Samples were centrifuged at 14,000 × g for 15 min at 4 °C, and the supernatant was transferred to LC vials for analysis.

Untargeted metabolomics was performed on an ultra-high-performance liquid chromatography (UHPLC) system (Vanquish, Thermo Fisher Scientific) coupled to a high-resolution Q Exactive Orbitrap mass spectrometer (Thermo Fisher Scientific) operated in both positive and negative electrospray ionization modes. Chromatographic separation was achieved on a C18 column (2.1 × 100 mm, 1.7 µm) with a water/acetonitrile gradient, both containing 0.1% formic acid.

Raw data were processed using Compound Discoverer or XCMS for peak picking, retention time alignment, and normalization. Multivariate analyses (PCA, PLS-DA), hierarchical clustering heatmaps, and variable importance in projection (VIP) scores were generated to identify discriminant metabolites between NC and diabetic groups. Metabolites were annotated by matching to in-house and public databases (HMDB, KEGG, mzCloud). Differential metabolites (VIP > 1.0, |log2FC| ≥ 0.58, adjusted P < 0.05) were subjected to KEGG pathway enrichment analysis, focusing on steroid hormone biosynthesis, arachidonic acid metabolism, and longevity-regulating pathways. Relative serum levels of PGA1 were extracted from the metabolomics dataset and further validated, if needed, by targeted LC–MS/MS.

**2.4 Tendon proteomics and integrative multi-omics analysis**

Fresh tendons from NC_T and DM_T mice (n = 3 per group) were pulverized in liquid nitrogen and lysed in 8 M urea buffer containing 1% SDS, protease and phosphatase inhibitors (Yeasen, Shanghai, China). Homogenates were sonicated on ice and centrifuged at 14,000 × g for 20 min at 4 °C to remove debris. Protein concentration in the supernatant was quantified using a BCA assay kit (Thermo Fisher Scientific).

Equal amounts of protein (e.g., 100 µg) were reduced with 10 mM dithiothreitol at 56 °C for 45 min, alkylated with 20 mM iodoacetamide in the dark at room temperature for 30 min, and digested overnight with sequencing-grade trypsin (Promega, USA; 1:50, w/w) after diluting urea to <1 M. Peptides were desalted using C18 cartridges and subjected to LC–MS/MS analysis on an EASY-nLC 1200 system coupled to a Q Exactive HF mass spectrometer (Thermo Fisher Scientific) in data-dependent acquisition mode.

MS/MS spectra were searched against the UniProt mouse database using MaxQuant (v2.x) with a 1% FDR at peptide and protein levels. Label-free quantification (LFQ) intensities were used for relative protein quantification. Differentially expressed proteins between NC_T and DM_T tendons were identified (|log2FC| ≥ 0.58, adjusted P < 0.05), and GO and GSEA enrichment analyses were performed to highlight ECM organization, inflammatory response and apoptosis pathways. Integrative analysis of scRNA-seq, metabolomics and proteomics data was performed to prioritize CD14 and PGA1-related signaling for mechanistic validation.

**3. Molecular modeling and binding validation**

The 3D structure of mouse CD14 was obtained from the Protein Data Bank, and the chemical structure of PGA1 was retrieved from PubChem and energy-minimized using Chem3D. Protein and ligand structures were prepared using AutoDockTools, with water molecules removed and Gasteiger charges assigned.

Molecular docking was performed using AutoDock Vina to predict the binding mode of PGA1 within the CD14 binding pocket. A grid box was defined to cover the putative ligand-binding region based on known CD14 ligands. The top-ranked pose, with the lowest binding free energy, was selected for further refinement.

The PGA1–CD14 complex underwent all-atom molecular dynamics (MD) simulations using GROMACS (v2021) with the CHARMM36m force field in explicit TIP3P water. The system was neutralized with counterions, energy-minimized, and equilibrated under NVT and NPT ensembles. Production runs (100 ns) were conducted at 310 K and 1 atm. Trajectories were analyzed for root mean square deviation (RMSD), root mean square fluctuation (RMSF), radius of gyration, solvent-accessible surface area, and hydrogen bonding between PGA1 and CD14. Free-energy landscapes and MM-PBSA/GBSA binding free energies were calculated to assess binding stability and identify key residues involved in ligand recognition.

**4. TSPC isolation and in vitro assays**

**4.1 Isolation and culture of tendon stem/progenitor cells (TSPCs)**

TSPCs were isolated from mouse tendon tissues as described previously with modifications^5,6^. Briefly, tendons from NC and diabetic mice were dissected aseptically, minced and digested with collagenase type I (2 mg/mL) and dispase II (2 mg/mL) in DMEM at 37 °C for 2–3 h with gentle shaking. The digested suspension was filtered through a 70 µm mesh, centrifuged at 300 × g for 5 min, and resuspended in PBS containing 2% FBS.

For flow cytometric sorting, cells were incubated on ice for 30 min with fluorochrome-conjugated antibodies against CD45, CD31 and Sca-1 (BioLegend, USA). After washing, CD45⁻CD31⁻Sca-1⁺ cells were sorted using a FACSAria III cell sorter (BD Biosciences) and collected in DMEM/F12 containing 10% FBS. Sorted TSPCs were plated on tissue culture flasks and expanded at 37 °C in 5% CO₂. Cells at passages 2–5 were used in all in vitro experiments.

**4.2 CD14 overexpression, siRNA-mediated CD14 knockdown and PGA1 treatment in TSPCs**

To generate CD14-overexpressing TSPCs (CD14^hi^ TSPCs), the full-length mouse CD14 cDNA (Gene ID: 12475) was cloned into a CMV-driven expression vector (pEX-5-CMV, GenScript, Nanjing, China). TSPCs were seeded in 6-well plates at 2 × 10⁵ cells/well and transfected with 1–2 µg plasmid per well using Lipofectamine 3000 (Thermo Fisher Scientific) according to the manufacturer’s instructions. Six hours after transfection, the medium was replaced with fresh complete medium. Stably overexpressing cell lines were selected with G418 (400 µg/mL; Sigma–Aldrich) for 10–14 days. CD14 overexpression was confirmed by qPCR and Western blotting.

For CD14 knockdown, TSPCs or CD14^hi^ TSPCs were transfected with siRNAs targeting Cd14 (si-CD14; GenePharma, Shanghai, China) using Lipofectamine RNAiMAX (Thermo Fisher Scientific). A non-targeting siRNA with no known homology to mouse transcripts was used as the negative control (si-Ctrl) to account for potential off-target effects of siRNA transfection. The final siRNA concentration was 20 nM. After 48 h, knockdown efficiency was assessed by qPCR and Western blotting before functional assays.

PGA1 (Sigma–Aldrich) was dissolved in DMSO to prepare a stock solution and diluted in culture medium to final concentrations of 1 and 10 µM (low and high doses) with 0.1% DMSO. Vehicle controls contained 0.1% DMSO without PGA1. CD14^hi^ TSPCs were treated with free PGA1, si-CD14, or their combinations for 24–48 h before functional and molecular analyses.

**4.3 Functional assays of TSPCs (proliferation, cell cycle, migration and apoptosis)**

***EdU incorporation assay:***

TSPCs or CD14^hi^ TSPCs were seeded in 24-well plates (1 × 10⁴ cells/well) and treated with vehicle, free PGA1, Lipo@PGA1 (low or high dose), si-CD14, or their combinations for 24 h. EdU (10 µM, Click-iT EdU Imaging Kit, Thermo Fisher) was added for the final 2 h. Cells were fixed with 4% paraformaldehyde, permeabilized with 0.5% Triton X-100, and subjected to the click reaction to label incorporated EdU. Nuclei were counterstained with DAPI. Images were acquired by fluorescence microscopy, and the percentage of EdU-positive cells was calculated as EdU⁺/DAPI⁺ × 100%.

***Cell cycle analysis:***

TSPCs were seeded on 6-well plates (2 × 10⁵ cells/well) and treated as indicated for 48 h. Cells were harvested, washed with cold PBS and fixed in 70% ethanol at −20 °C overnight. After washing, cells were resuspended in PBS containing 50 µg/mL PI and 100 µg/mL RNase A, and incubated at room temperature in the dark for 30 min. DNA content was analyzed using a flow cytometer (BD FACSCalibur, BD Biosciences). The percentages of cells in G0/G1, S and G2/M phases were determined with FlowJo (v10.0).

***Scratch-wound migration assay:***

TSPCs were seeded into 6-well plates and grown to confluence. A linear wound was created using a sterile 200 µL pipette tip. Detached cells were removed by PBS washing, and cells were cultured in serum-reduced medium (1% FBS) with the indicated treatments. Images of the same regions were taken at 0 and 24 h under phase-contrast microscopy. Wound closure (%) was quantified as (initial gap area – final gap area)/initial gap area × 100% using ImageJ.

***Annexin V/PI apoptosis and live/dead staining:***

To quantify apoptosis, TSPCs were treated as above for 24 h, harvested, washed twice with cold PBS, and resuspended in Annexin V binding buffer. Annexin V-FITC and PI (Beyotime) were added, and cells were incubated for 15 min at room temperature in the dark. Samples were analyzed by flow cytometry (BD FACSCalibur). The proportions of viable (Annexin V⁻/PI⁻), early apoptotic (Annexin V⁺/PI⁻), late apoptotic/necrotic (Annexin V⁺/PI⁺) and dead (Annexin V⁻/PI⁺) cells were calculated. Live/dead staining of TSPCs was performed as described for BMSCs.

**5. Liposome preparation and characterization**

**5.1 Preparation and characterization of PGA1-loaded liposomes (Lipo@PGA1)**

PGA1-loaded liposomes were prepared using a thin-film hydration method. Briefly, soy phosphatidylcholine (S100, Lipoid, Germany), cholesterol (Sigma–Aldrich) and DSPE-PEG2000 (Avanti Polar Lipids, USA) were dissolved in chloroform/methanol (2:1, v/v) at a molar ratio of 65:30:5 in a round-bottom flask. The organic solvent was evaporated under reduced pressure using a rotary evaporator to form a thin lipid film, followed by vacuum desiccation for at least 4 h to remove residual solvent.

For Lipo@PGA1, PGA1 was dissolved in a small volume of ethanol and added to the lipid mixture before film formation (final concentrations of 1 and 10 µM-low and high doses). The dry film was hydrated with PBS (pH 7.4) at 37 °C for 30 min under gentle rotation to obtain multilamellar vesicles, which were then extruded sequentially through 200 and 100 nm polycarbonate membranes (Whatman, USA) to generate nanoscale unilamellar vesicles. Blank liposomes (Lipo@Ctrl) were prepared using the same protocol without PGA1.

Hydrodynamic diameter, polydispersity index (PDI) and zeta potential of liposomes were measured by dynamic light scattering (Zetasizer Nano-ZS, Malvern, UK) at 25 °C. The morphology of blank liposomes and Lipo@PGA1 was observed by transmission electron microscopy (TEM; JEM-2100, JEOL, Japan) and scanning electron microscopy (SEM; SU8200, Hitachi, Japan) after negative staining and lyophilization, respectively. PGA1 loading content and encapsulation efficiency were quantified by HPLC after disrupting liposomes with methanol. In vitro PGA1 release was evaluated under sink conditions at 37 °C. Free PGA1 solution and Lipo@PGA1 were dispersed in PBS (pH 7.4) or PBS containing 10% FBS (or acetate buffer, pH 6.5) and placed in a shaking water bath. At predetermined time points (0, 1, 2, 4, 8, 12, 24, 48, and 72 h), aliquots of the release medium were collected and replaced with fresh buffer. PGA1 concentrations were quantified by HPLC, and cumulative release was calculated and expressed as a percentage of the total PGA1 loading.

**5.2 In vivo near-infrared fluorescent tracking of DiR-labeled liposomes**

To evaluate the local retention of liposomes at the tendon–bone interface, we prepared DiR-labeled PGA1-loaded liposomes (DiR-Lipo@PGA1) and a free DiR control. The near-infrared lipophilic dye DiR (1,1'-dioctadecyl-3,3,3',3'-tetramethylindotricarbocyanine iodide; Beyotime, Shanghai, China) was incorporated into the lipid phase at 0.5 mol% of total lipid during liposome preparation. DiR-Lipo@PGA1 was obtained following the same procedure as Lipo@PGA1, with DiR added to the ethanolic lipid mixture before nanoparticle formation. For the free DiR group, DiR was dissolved in a mixture of ethanol/PEG-400/saline (5:5:90, v/v/v) and diluted to match the DiR dose used in the liposomal formulation.

After establishment of the diabetic supraspinatus RCT model and surgical repair, rats were randomly assigned to receive peri-shoulder injections of either DiR-Lipo@PGA1 or free DiR (100 μL per shoulder, equivalent DiR dose per rat). Injections were administered around the tendon–bone interface immediately after repair. Whole-body near-infrared fluorescence imaging was performed using an in vivo imaging system (IVIS Spectrum, PerkinElmer, USA) at 0, 4, 8, 24 and 48 h post-injection. Images were acquired with an excitation filter of 748 nm and an emission filter of 780 nm under identical exposure settings. Regions of interest (ROIs) were drawn around the operated shoulder, and total radiant efficiency (photons/s/cm²/sr)/(μW/cm²) was quantified using Living Image software (v4.5). Fluorescence intensity was normalized to the 0-h value for each animal.

**5.3 In vivo administration of liposomes and biosafety evaluation**

For local delivery, Lipo@PGA1 or blank liposomes were diluted in sterile PBS and injected peri-tendon around the supraspinatus tendon–bone interface (100 µL liposomes per shoulder) immediately after RCT repair and at predefined postoperative time points under brief anesthesia.

To evaluate systemic biosafety, rats receiving repeated peri-shoulder injections of Lipo@PGA1 were euthanized at the endpoint. Heart, liver, spleen, lung and kidney were harvested, fixed in 4% paraformaldehyde for at least 24 h, embedded in paraffin, and sectioned at 5 µm. Sections were stained with hematoxylin and eosin (H&E) and Masson’s trichrome following standard protocols to assess tissue architecture, fibrosis and inflammatory infiltrates. No gross or microscopic abnormalities were observed in major organs as shown in Fig. 6 and Supplementary Fig. 7.

**6. In vivo rotator cuff repair (RCT) repair and functional evaluation**

**6.1 Diabetic rat supraspinatus RCT model and liposome treatment**

After confirmation of T2DM, rats were randomly assigned to four groups: Diabetes-NC (sham surgery), RCT (diabetic RCT repair without liposomes), Lipo@Ctrl (RCT repair with blank liposomes), and Lipo@PGA1 (RCT repair with PGA1-loaded liposomes).

Under general anesthesia with 5% pentobarbital sodium (45 mg/kg, i.p.), the right shoulder region was shaved and disinfected. A longitudinal skin incision was made along the lateral aspect of the shoulder, and the deltoid muscle was split to expose the supraspinatus tendon. The tendon was sharply detached from the greater tuberosity, and the footprint was lightly decorticated. A transosseous tunnel was drilled through the greater tuberosity with a 1.0 mm Kirschner wire. The supraspinatus tendon was reattached using a modified Mason–Allen suture passed through the bone tunnel. Immediately after repair, 100–200 µL of Lipo@PGA1 or Lipo@Ctrl was injected around the tendon–bone interface with a microsyringe. The deltoid and skin were closed in layers.

The Diabetes-NC group underwent exposure without tendon detachment, whereas the RCT group underwent RCT repair without liposomes. Postoperatively, rats were allowed free cage activity without immobilization and received analgesia (meloxicam 1 mg/kg, s.c.) for 3 days. The shoulder joint was monitored daily for wound healing and infection.

**6.2 Histology and semi-quantitative scoring of tendon–bone healing**

At 4 and 8 weeks after surgery, rats were euthanized, and supraspinatus tendon–humerus complexes were harvested. Specimens were fixed in 4% paraformaldehyde, decalcified in 10% EDTA (pH 7.4) at 4 °C for 4–6 weeks with frequent solution changes, dehydrated, embedded in paraffin and sectioned in the sagittal plane (5 µm).

Sections were stained with H&E to evaluate general morphology and continuity of the tendon–bone interface and with Masson’s trichrome to assess collagen fiber organization. Safranin O–fast green staining was performed to visualize fibrocartilage and proteoglycan deposition. Histological images were captured using an upright microscope (Olympus BX53, Japan).

Semi-quantitative histological scores were assigned by two blinded observers using a modified tendon–bone healing scoring system according to our previous study, evaluating parameters such as interface continuity, fibrocartilage formation, collagen fiber orientation and cellularity^5,6^. Scores from different groups and time points were compared statistically.

**6.3 Transmission electron microscopy (TEM) of regenerated tendon**

For ultrastructural evaluation, small blocks (~1 mm³) of regenerated tendon at the insertion site were fixed in 2.5% glutaraldehyde in 0.1 M phosphate buffer (pH 7.4) at 4 °C overnight, post-fixed in 1% osmium tetroxide for 1–2 h, dehydrated in graded ethanol, and embedded in epoxy resin. Ultrathin sections (70–90 nm) were cut with an ultramicrotome, stained with uranyl acetate and lead citrate, and examined under a transmission electron microscope (JEM-1230, JEOL). Collagen fibril diameter, density and packing arrangement were quantified using ImageJ or Origin software from multiple randomly selected fields per sample.

**6.4 Biomechanical testing of supraspinatus tendon–humerus complexes**

At 8 weeks, supraspinatus tendon–humerus units were carefully dissected free of surrounding tissues while preserving the tendon insertion. The humerus was potted in polymethylmethacrylate, and the supraspinatus tendon was clamped in a custom fixture. Specimens were mounted on a universal testing machine (Instron 5943, USA) with the tendon oriented along the loading axis.

A small preload (0.1–0.2 N) was applied for 3 min to remove slack, followed by uniaxial tensile loading at a constant displacement rate of 2 mm/min until failure. Load–displacement curves were recorded, and maximum load to failure (N) and stiffness (N/mm, calculated from the linear region of the curve) were determined. Ultimate stress (MPa) was calculated by normalizing maximum load to cross-sectional area when feasible.

**6.5 Gait analysis and forelimb grip-strength assessment**

Gait analysis was performed using the CatWalk XT system (Noldus, Netherlands) at 8 weeks postoperatively. Rats were trained to traverse the glass walkway for at least 3 days before data collection. At each session, rats were allowed to walk freely across the walkway until three valid runs (run duration 2–10 s, speed variation ≤40%, no turning) were recorded. Paw prints were captured by the high-speed camera and analyzed using CatWalk XT 10.0 software. Parameters including paw pressure, print area and swing speed of the operated forelimb were extracted and averaged per animal.

Forelimb grip strength of the operated limb was measured using a digital grip-strength meter (XR501, Shanghai Xinrun, China). Rats were allowed to grasp a horizontal bar with their forepaws while being gently pulled backward until they released the bar. Peak force (N) was recorded. Each rat performed three consecutive trials with 2 min rest intervals, and the mean value was used for analysis.

**7. Molecular and histological assays**

**7.1 Western blot analysis**

Total protein was extracted from cultured TSPCs or tendon–bone insertion tissues using RIPA lysis buffer supplemented with protease and phosphatase inhibitors (Yeasen, Shanghai, China)^7–9^. Lysates were incubated on ice for 15 min and centrifuged at 12,000 × g for 15 min at 4 °C. Protein concentration was measured by BCA assay.

Equal amounts of protein (20–30 µg) were separated on 8–12% SDS–PAGE gels and transferred to PVDF membranes (Millipore). Membranes were blocked with 5% non-fat milk in TBST for 1 h at room temperature and incubated overnight at 4 °C with primary antibodies against CD14, IκBα, phospho-IκBα, NF-κB p65, phospho-p65, Bax, Bcl-2, cleaved caspase-3, collagen I, collagen III, GAPDH and β-tubulin (1:1,000, Abcam, Cell Signaling Technology or Affinity). After washing, membranes were incubated with HRP-conjugated secondary antibodies (1:5,000) for 1 h at room temperature. Bands were visualized using ECL reagents (Thermo Fisher Scientific) and imaged with a ChemiDoc MP system (Bio-Rad). Band intensities were quantified by ImageJ and normalized to GAPDH or β-tubulin.

**7.2 Immunofluorescence staining of cells and tendon–bone interface tissues**

For cellular immunofluorescence, TSPCs were seeded on glass coverslips in 24-well plates and treated as indicated^10,11^. Cells were fixed with 4% paraformaldehyde for 10 min, washed with PBS, permeabilized with 0.1–0.3% Triton X-100 for 10 min, and blocked with 5% BSA in PBS for 1 h at room temperature. Cells were incubated overnight at 4 °C with primary antibodies against CD14, NF-κB p65, CD44, TNMD or α-SMA, followed by Alexa Fluor 488- or 594-conjugated secondary antibodies (1:500; Abcam) for 1 h at room temperature in the dark. F-actin was stained with Alexa Fluor 488–phalloidin when required. Nuclei were counterstained with DAPI. Images were acquired using a confocal microscope (Leica, Germany). Nuclear/cytoplasmic NF-κB localization and fluorescence intensities were quantified using ImageJ.

For tissue immunofluorescence, decalcified supraspinatus tendon–humerus complexes were embedded in paraffin and sectioned at 5 µm. After deparaffinization and rehydration, antigen retrieval was performed in citrate buffer (pH 6.0) using a microwave or steamer. Sections were blocked with 5% BSA/0.5% Triton X-100 for 1 h and incubated overnight at 4 °C with primary antibodies against collagen I, collagen III, Sox9, α-SMA, CD44 and TNMD. After washing, sections were incubated with appropriate Alexa Fluor–conjugated secondary antibodies for 1 h. Nuclei were stained with DAPI. Images were obtained using a fluorescence microscope (Leica DMi8), and positive cell counts or fluorescence intensities were quantified in regions of interest at the tendon–bone interface.

**7.3 Quantitative real-time PCR (qPCR)**

Total RNA was extracted from cultured TSPCs or tendon–bone insertion tissues using TRIzol reagent (Invitrogen, USA) according to the manufacturer’s protocol. RNA purity and concentration were assessed by NanoDrop 2000 (Thermo Scientific). cDNA was synthesized using a PrimeScript RT Reagent Kit (Takara, Japan). qPCR was performed using TB Green Premix Ex Taq II (Takara) on a CFX96 real-time PCR system (Bio-Rad, USA).

Relative mRNA levels of target genes (Cd14, Bax, Bcl-2, Caspase-3, inflammatory cytokines, matrix markers, etc.) were calculated using the 2^−ΔΔCt^ method, with Gapdh as the internal control. Each sample was analyzed in triplicate.

**8. Statistical analysis**

All experiments were repeated three times independently. Data are presented as mean ± standard deviation (SD) unless otherwise indicated. Statistical analyses were performed using GraphPad Prism 9.0 (GraphPad Software, CA, USA). For comparisons between two groups, unpaired Student’s t-test or Mann–Whitney U test was applied depending on data distribution. For comparisons among multiple groups, one-way or two-way ANOVA followed by Bonferroni’s post hoc test was used.

Normality and variance homogeneity were assessed before parametric testing. For multiple-group comparisons, one-way ANOVA with post hoc correction was used when assumptions were met; otherwise, Welch’s ANOVA or non-parametric alternatives were applied. Multiple testing correction for omics analyses was performed as described in the corresponding methods sections.

For scRNA-seq, metabolomics and proteomics datasets, multiple testing correction was performed using the Benjamini–Hochberg method, and an adjusted P < 0.05 was considered statistically significant. All P values were two-tailed, and P < 0.05, P < 0.01, P < 0.001 and P < 0.0001 were interpreted as significant, highly significant and extremely significant, respectively.

**Sup Figures:**

**Supplementary Figure 1. Validation of the type 2 diabetes mouse model.**

(a) Body weight of normoglycemic control (NC) and diabetic mice at the end of the induction period. Diabetic mice displayed significantly higher body weight than NC mice.

(b) Fasting blood glucose (FBG) levels in NC and diabetic mice at the same time point, showing a marked elevation of FBG in the diabetic group.

Both readouts confirm successful establishment of the type 2 diabetes model. Data are presented as mean ± SD (n = 5 mice per group), with individual animals overlaid as scatter points; **P** < 0.0001 for NC vs. Diabetes in both panels.

**Supplementary Figure 2. Quality control metrics of single-cell RNA sequencing data.**

(a) Scatter plots of quality control (QC) metrics for all captured cells, showing the relationships between total UMI counts and number of detected genes (left), percentage of mitochondrial transcripts and number of genes (middle), and percentage of mitochondrial transcripts and total counts (right). Dashed lines indicate the filtering thresholds applied for downstream analysis (200–6000 genes per cell, 100–100,000 total counts per cell, and <30% mitochondrial counts).

(b) Violin plots summarizing the global distribution of the number of genes, total counts and mitochondrial transcript percentages across all cells after QC filtering, demonstrating that the majority of cells fall within the predefined thresholds.

(c) Group-wise violin plots of the same QC parameters (number of genes, total counts and mitochondrial percentage) for each sample group (DM_T, NC_T and OP_T), indicating comparable data quality and library complexity among samples included in the integrated single-cell analysis.

**Supplementary Figure 3. Marker-gene heatmap used for single-cell cluster annotation and pathway enrichment analysis of diabetic tendon cells.**

(a) Heatmap of representative canonical tendon and stromal marker genes used for cluster identification across tendon-derived single-cell clusters. Rows represent selected marker genes and columns represent single-cell clusters; color intensity indicates normalized expression levels. This marker-gene heatmap was used to support the assignment of major tendon-resident cell populations, including TSPC-enriched progenitor-like stromal cells, fibroblasts, endothelial cells, immune cells and other stromal populations.

(b) Network plots of Gene Ontology (GO) biological process (BP), molecular function (MF), cellular component (CC) and KEGG terms enriched in differentially expressed genes from diabetic TSPCs. Nodes represent genes (circles) and enriched terms (squares), with edges indicating gene–term relationships, highlighting pathways related to extracellular matrix organization, collagen fibril formation, integrin signaling and focal adhesion.

(c) Bubble plots summarizing pathway enrichment analyses of diabetic versus control TSPCs. Shown are GO BP, GO MF, GO CC, KEGG, Reactome and WikiPathways (WP) terms, with dot size proportional to the number of genes in each term and color reflecting adjusted *P* values. Enriched pathways emphasize ECM–receptor interaction, collagen biosynthesis, focal adhesion and other matrix-remodeling and cell-adhesion processes associated with TSPC dysfunction in diabetes.

**Supplementary Figure 4. In vitro biosafety evaluation of liposomal PGA1 in human BMSCs.**

(a) Quantification of DA fluorescence intensity per area in human bone marrow–derived mesenchymal stem cells (BMSCs) treated with vehicle (NC), free PGA1, blank liposomes (Lipo@Ctrl) or PGA1-loaded liposomes (Lipo@PGA1), showing no significant increase in DA signal among the treated groups, indicating absence of detectable cytotoxic/oxidative stress effects.

(b) BrdU incorporation assay of BMSCs under the same treatments, with the number of BrdU-positive nuclei per area demonstrating that neither free PGA1 nor liposomal formulations impair BMSC proliferative capacity.

Together, these data suggest that Lipo@PGA1 exhibits good in vitro biocompatibility in human BMSCs.

**Supplementary Fig. 5 | In vitro release profiles of PGA1 from free drug and liposomal formulations under different media conditions.**

Cumulative release of PGA1 (%) from free PGA1 solution and Lipo@PGA1 incubated in PBS (pH 7.4), PBS containing 10% fetal bovine serum (FBS), or acetate buffer (pH 6.5) at 37 °C over 72 h (n = 3, mean ± SD). Free PGA1 showed rapid diffusion with nearly complete release within 4 h, whereashen Lipo@PGA1 exhibited a sustained and medium-dependent release profile.

**Supplementary Figure 6. Validation of the type 2 diabetes rat model.**

(a) Body weight of normoglycemic control (NC) and diabetic rats at the end of the induction period. Diabetic rats exhibited significantly higher body weight than NC rats (*P* = 0.0016).

(b) Fasting blood glucose (FBG) levels in NC and diabetic rats at the same time point, showing a marked elevation of FBG in the diabetic group (*P* < 0.0001).

Data are presented as mean ± SD (n = 5 rats per group), with individual animals overlaid as scatter points, confirming successful establishment of the type 2 diabetes rat model.

**Supplementary Figure 7. In vivo biosafety evaluation of liposomal PGA1 in rats by multi-organ Masson’s trichrome staining.**

Representative Masson’s trichrome–stained sections of heart, liver, spleen, lung and kidney harvested from rats in the different treatment groups (e.g., NC, Lipo@Ctrl and Lipo@PGA1). Across all organs and treatment conditions, myocardial fibers, hepatic lobules, splenic parenchyma, alveolar structures and renal glomeruli/tubules display preserved architecture without obvious collagen over-deposition, inflammatory infiltration or tissue necrosis, indicating that repeated administration of Lipo@PGA1 does not induce detectable fibrosis or structural toxicity in major organs. Scale bars, 200 μm.

**Supplementary Figure 8. Histological scoring of tendon–bone healing among treatment groups.**

(a) Semi-quantitative histological scores of the supraspinatus tendon–bone interface in the Diabetes-NC, RCT, Lipo@Ctrl and Lipo@PGA1 groups. Diabetic RCT tendons show markedly higher (worse) histological scores compared with treated groups, whereas Lipo@PGA1 treatment significantly improves the histological appearance of the repair site relative to both untreated RCT and Lipo@Ctrl groups, indicating superior tissue regeneration. Data are presented as mean ± SD (n = 5 per group); corresponding *P* values are indicated above the bars.

**Supplementary Figure 9. Western blot analysis of CD14 expression in supraspinatus tendon–bone interface tissues.**

(a-b) Representative Western blot images showing CD14 protein levels (upper panel, ~40 kDa) in the Diabetes-NC, RCT, Lipo@Ctrl and Lipo@PGA1 groups, with Tubulin used as a loading control (lower panel). CD14 expression is markedly increased in the RCT group compared with Diabetes-NC, partially reduced in the Lipo@Ctrl group, and most prominently downregulated in the Lipo@PGA1 group, indicating that local delivery of PGA1-loaded liposomes effectively suppresses diabetes- and injury-associated upregulation of CD14 at the tendon–bone interface. Data are presented as mean ± SD (n = 3 per group); corresponding *P* values are indicated above the bars.

**References:**

1.	Liu, S. *et al.* Enhancing diabetic muscle repair through W-GA nanodots: a nanomedicinal approach to ameliorate myopathy in type 2 diabetes. *Burns Trauma* 13, (2025).

2.	Wang, Q., Huang, M. & Guo, J. J. From cells to clinic: Single-cell transcriptomics shaping the future of orthopedics. *J Orthop Translat* 53, 1–11 (2025).

3.	Chen, Y. *et al.* Exercise Modifies the Transcriptional Regulatory Features of Monocytes in Alzheimer’s Patients: A Multi-Omics Integration Analysis Based on Single Cell Technology. *Front Aging Neurosci* 14, 881488 (2022).

4.	Gu, X. *et al.* Identification and validation of a muscle failure index to predict prognosis and immunotherapy in lung adenocarcinoma through integrated analysis of bulk and single-cell RNA sequencing data. *Front Immunol* 13, 1–16 (2023).

5.	Qian, Y. *et al.* Bioactive siRNA-Based Liposomes Promoted Tendon-Bone Healing in Osteoporotic Mice by Recovering the Stemness of CD248+ TSPCs. *Advanced Science* 12, (2025).

6.	He, Y. *et al.* SHED-derived exosome-mimetics promotes rotator cuff tendon-bone healing via macrophage immunomodulation through NF-κB suppression and autophagy activation. *Mater Today Bio* 34, (2025).

7.	Lin, J. *et al.* Long non‐coding RNA H19 promotes myoblast fibrogenesis via regulating the miR‐20a‐5p‐Tgfbr2 axis. *Clin Exp Pharmacol Physiol* 48, 921–931 (2021).

8.	Sun, Y. *et al.* si-Tgfbr1-loading liposomes inhibit shoulder capsule fibrosis via mimicking the protective function of exosomes from patients with adhesive capsulitis. *Biomater Res* 26, 39 (2022).

9.	Luo, Z. *et al.* Human bone marrow mesenchymal stem cell-derived extracellular vesicles inhibit shoulder stiffness via let-7a/Tgfbr1 axis. *Bioact Mater* 17, 344–359 (2022).

10.	Sun, Y., Lin, J., Luo, Z., Zhang, Y. & Chen, J. The Serum from Patients with Secondary Frozen Shoulder Following Rotator Cuff Repair Induces Shoulder Capsule Fibrosis and Promotes Macrophage Polarization and Fibroblast Activation. *J Inflamm Res* Volume 14, 1055–1068 (2021).

11.	Luo, Z., Lin, J., Sun, Y., Wang, C. & Chen, J. Bone Marrow Stromal Cell-Derived Exosomes Promote Muscle Healing Following Contusion Through Macrophage Polarization. *Stem Cells Dev* 30, 135–148 (2021).
